# A membrane intercalating metal-free conjugated organic photosensitizer for bacterial photodynamic inactivation†

**DOI:** 10.1039/d3sc01168b

**Published:** 2023-07-05

**Authors:** Arianna Magni, Sara Mattiello, Luca Beverina, Giuseppe Mattioli, Matteo Moschetta, Anita Zucchi, Giuseppe Maria Paternò, Guglielmo Lanzani

**Affiliations:** a Department of Physics, Politecnico di Milano 20133 Milan Italy giuseppemaria.paterno@polimi.it; b Department of Materials Science, University of Milano-Bicocca 20125 Milan Italy; c CNR – Istituto di Struttura della Materia I-00015 Monterotondo Scalo Italy; d Center for Nano Science and Technology @PoliMi, Istituto Italiano di Tecnologia 20133 Milan Italy guglielmo.lanzani@iit.it

## Abstract

Photodynamic inhibition (PDI) of bacteria represents a powerful strategy for dealing with multidrug-resistant pathogens and infections, as it exhibits minimal development of antibiotic resistance. The PDI action stems from the generation of a triplet state in the photosensitizer (PS), which subsequently transfers energy or electrons to molecular oxygen, resulting in the formation of reactive oxygen species (ROS). These ROS are then able to damage cells, eventually causing bacterial eradication. Enhancing the efficacy of PDI includes the introduction of heavy atoms to augment triplet generation in the PS, as well as membrane intercalation to circumvent the problem of the short lifetime of ROS. However, the former approach can pose safety and environmental concerns, while achieving stable membrane partitioning remains challenging due to the complex outer envelope of bacteria. Here, we introduce a novel PS, consisting of a metal-free donor–acceptor thiophene-based conjugate molecule (BV-1). It presents several advantageous features for achieving effective PDI, namely: (i) it exhibits strong light absorption due to the conjugated donor–acceptor moieties; (ii) it exhibits spontaneous and stable membrane partitioning thanks to its amphiphilicity, accompanied by a strong fluorescence turn-on; (iii) it undergoes metal-free intersystem crossing, which occurs preferentially when the molecule resides in the membrane. All these properties, which we rationalized *via* optical spectroscopies and calculations, enable the effective eradication of *Escherichia coli*, with an inhibition concentration that is below that of current state-of-the-art treatments. Our approach holds significant potential for the development of new PS for controlling bacterial infections, particularly those caused by Gram-negative bacteria.

## Abstract 2

The molecule BV-1 is designed to target the bacterial membrane and produce reactive oxygen species at this location, leading to *E. coli* photo-inhibition at a relatively low minimum inhibition concentration.

## Graphical abstract

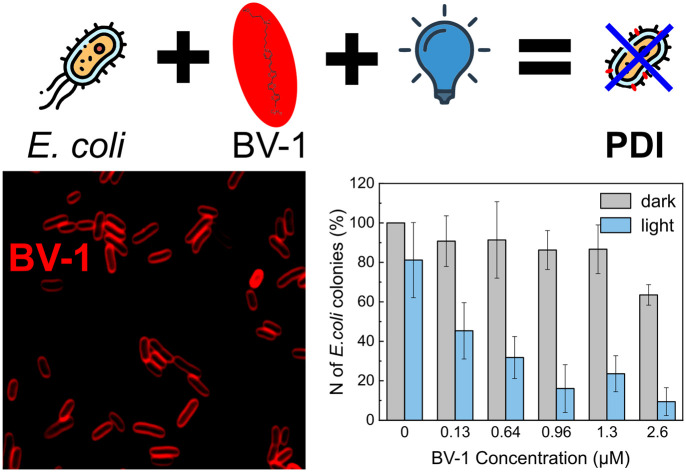

## Introduction

The excessive and, in some cases, inappropriate use of antibiotics in outpatient clinics, by hospitalized patients and in the food industry has resulted in the development of numerous antibiotic-resistant bacterial strains.^1–3^ Therefore, there is a pressing need to explore alternative bactericidal strategies that transcend conventional approaches.^4,5^ One possibility is the photodynamic inhibition or photodynamic inactivation (PDI) of pathogenic bacteria, in which the oxidative stress following irradiation with visible light leads to microbial death.^6^ PDI operates through the synergistic action of molecular oxygen, naturally present in cells, appropriately designed non-toxic photosensitizers (PS) and light. Upon light excitation, the PS ends up in a triplet state that can transfer energy/electrons to molecular oxygen. This process results in the formation of reactive oxygen species (ROS) or singlet oxygen radicals, which oxidise biomolecules such as lipids, proteins and nucleic acids,^7–9^ ultimately leading to cellular death and bacterial eradication. The major advantage of PDI lies in its limited potential for the development of resistance in microorganisms,^10,11^ holding promise for tackling antibiotic resistance in Gram-negative bacteria, against which no new class of antibiotics has been approved since the late 1980s.^12^

Most of the research efforts point toward the development of new and effective PS, exhibiting strong light absorption and emission features, enabling imaging-guided PDI,^13–15^ as well as enhanced intersystem crossing (ISC) from singlet to triplet states. The introduction of heavy atoms is usually employed as an efficient strategy to promote spin–orbit coupling and increase the population of the triplet state.^16–19^ However, this approach generally leads to quenching of the emission and poses toxicity and environmental issues. Another important challenge to face is the relatively short lifetime of ROS, as they easily react with biomolecules close to the generation site. Membrane-intercalating PS are considered an effective tool to circumvent this problem,^20^ as the proximity between the PS and cells would compensate for the short lifetime of the reactive species. This aspect is extremely relevant for bacteria, as in this case, the membrane is the core of their bioenergetics since the synthesis of ATP occurs at this location,^21–23^ thus representing an ideal biological target for PDI purposes.

In this study, we report a newly synthesized organic PS, named BV-1, which is a metal-free donor–acceptor thiophene-based conjugate molecule. This system affords a series of advantageous aspects for PDI, thanks to the precise engineering of its molecular features and excited state pathways, including: (i) strong optical absorption in the visible range due to π-conjugation and the donor–acceptor moiety; (ii) non-covalent affinity for the lipid membrane, facilitated by its amphiphilicity, permitting stable and spontaneous partitioning within the membrane. Notably, membrane intercalation is accompanied by a strong fluorescence turn-on, thanks to the suppression of non-radiative conformational relaxation processes; (iii) metal-free ISC, a process that is favoured within the membrane environment as it inhibits competitive and rapid deactivation of the first excited state with charge-transfer character. These properties, which were investigated in detail and rationalised through ultrafast optical spectroscopies and calculations, lead to effective light inactivation of *E. coli* at a minimum inhibitory concentration (0.12 μM) which is below the state-of-the-art level (at least one order of magnitude) for Gram-negative bacteria^19,24^ thereby highlighting its potential for broad-spectrum PDI applications.^25^

## Results and discussion

### Synthesis of BV-1 and molecular rationale

**Scheme 1:**
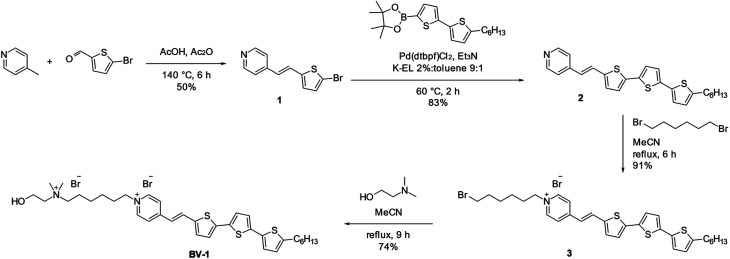
Synthetic route to BV-1. The reaction pathway followed to synthesize BV-1.

The molecule, named BV-1, was synthesised according to the reaction route reported in Scheme 1. Full details about the synthesis and NMR characterisation can be found in the Materials section and Fig. S1–S7.† The BV-1 molecular structure (Scheme 1) contains three blocks with different functions: (i) a π-conjugated electron donor–acceptor pair consisting of a terthiophene and a pyridinium moiety conjugated through a *trans* –C=C– bridge, which provides strong optical absorption/emission in the visible range; (ii) an alkyl chain possessing a non-covalent affinity to the lipophilic core of the lipid membrane that can interact with the phospholipid tails; (iii) a hydrophilic head consisting of a quaternary ammonium cation and a hydroxyl-terminated unit, ensuring solubility in saline solution and with a strong capability to interact with the phospholipid heads.

### BV-1 association with the bacterial membrane

**Fig. 1:**
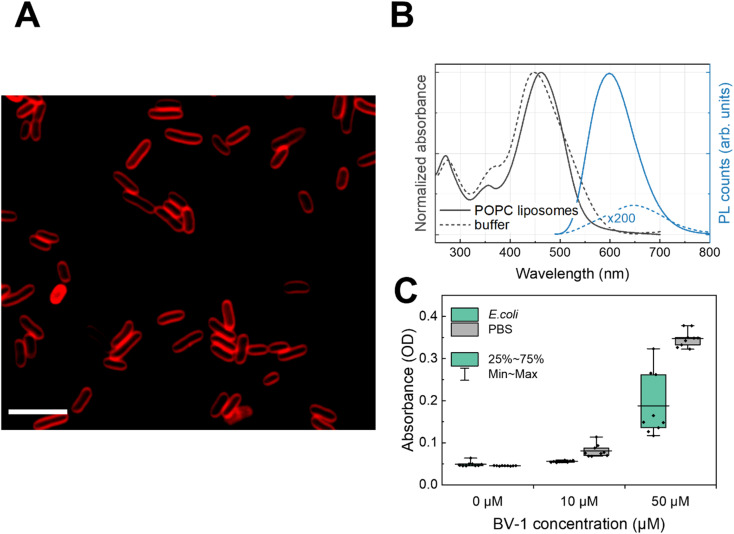
(A) Confocal fluorescence image of the *E. coli* culture exposed to BV-1 [2 μM] to evaluate the membrane incorporation of BV-1. Scale bar 5 μm. (B) Absorbance and PL spectra of BV-1 [25 μM] in POPC liposomes suspension (solid line) and in its aqueous buffer solution (dashed lines). (C) Cell association (uptake) of BV-1 with *E. coli* cells. Samples were stained with BV-1 at the specified concentrations and maintained at 37 °C for 60 minutes in the dark. The absorbance of the supernatant was measured at 470 nm. Control samples (PBS buffer) with no cells were treated the same and their absorbance values represented the total as a reference. All conditions and controls were measured in triplicate.

First, we exploited the fluorescence properties of the π-conjugated block of BV-1 to visualise its localization within the *E. coli* cells *via* fluorescence microscopy. Confocal imaging (Fig. 1A) shows that after incubation with the molecule, bacterial cells display an emission that is well localized around the edges of cells, which is consistent with membrane intercalation of BV-1. We also took advantage of the relatively strong absorption of BV-1 to quantify the amount of molecules associated with *E. coli* cells. This was evaluated by measuring the absorbance of a fixed amount of BV-1 solution before and after 1 h of incubation in *E. coli* (Fig. 1C). The data show that more than 50% of the molecule is associated with the cells. Starting from an initial concentration of 10 μM of BV-1 in the solution, the bacteria cells take up around 80% of the molecules, while increasing the initial concentration up to 50 μM leads to an uptake of 53%. This drop is likely related to the saturation of the amount of BV-1 that can be stored in the membrane of *E. coli* cells. The association of the dye with the membrane of *E. coli* is stable over 24 hours, as shown in Fig. S18.†

Characterizing the spectroscopic properties of molecules in living cells is challenging. We therefore studied the static absorption/emission features of the BV-1 in artificial lipid bilayers, which mimic the plasma membrane. By comparing the spectra of the molecule in the liposome suspension with the buffer solution (Fig. 1B), we further proved the affinity of BV-1 for the biological membranes. Specifically, we employed 1-palmitoyl-2-oleoyl-*sn*-glycero-3-phosphocholine (POPC) liposomes, suspended at a concentration of 1 mg mL^−1^ in an aqueous buffer solution (10 mM tris(hydroxymethyl)aminomethane, 100 mM NaCl, pH 7.4). Starting with the absorption, we observed a ∼10 nm shift in POPC liposomes compared to the buffer solution, suggesting a strong interaction of BV-1 with the lipid bilayer. Moving on to the photoluminescence (PL), we noticed a 50 nm blue shift when the molecule associates with the lipid bilayer compared to the buffer solution, accompanied by a ∼1000-fold increase in the PL quantum yield (PLQY) (see later for more details). These data, taken together, confirm the spontaneous partitioning of BV-1 into the cell membrane, primarily driven by its amphiphilicity.

### BV-1 steady-state photophysics

Initially, we investigated the linear absorption and emission properties of BV-1 as a function of solvent polarity (solvatochromism). The objective was to acquire fundamental insights into the potential photophysical pathways undergone by BV-1, including charge-transfer transitions, singlet deactivation and ISC. With this in mind, we employed different solvents with dielectric constants spanning approximately one order of magnitude. Specifically, we utilized dichloromethane (DCM, *ε* = 8.93), ethanol (EtOH, *ε* = 24.5), and water (*ε* = 80.1).

**Fig. 2:**
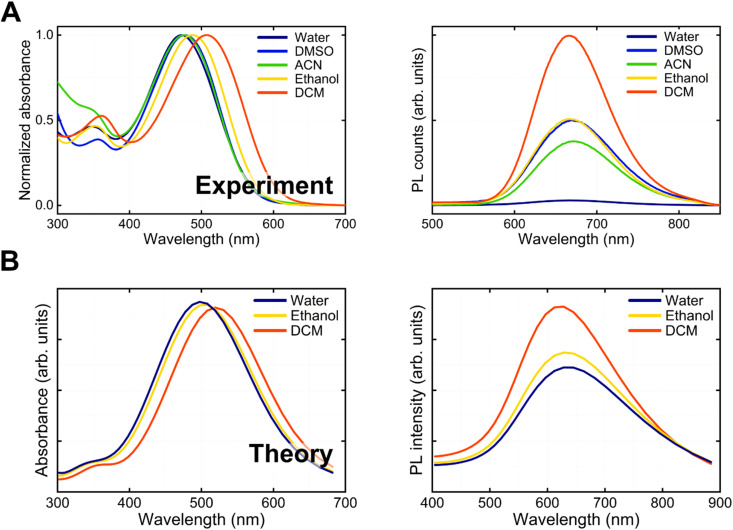
(A) Normalized absorption spectra (left) and emission spectra (right) of BV-1 (25 μM) in different solvents. (B) Absorption and PL spectra of BV-1 in a few solvents (dichloromethane, ethanol and water) as calculated by TDDFT.

We started looking at ground state absorption (Fig. 2A), observing a negative (hypsochromic) solvatochromism, with the main absorption peak shifting from 507 nm in DCM to 470 nm in water. The strong absorption of BV-1 in the visible range corresponds to an excited state having a charge transfer character, with charge density moving from the donor terthiophene chain to the acceptor pyridinium unit. This conclusion was further substantiated by simulations (Fig. S8†). The solvatochromism is a consequence of the polar nature of the ground state, which shifts to lower energy in the presence of solvents of increasing polarity. Accordingly, the blue-shift of the absorption peak is larger for saline water solutions of increasing ionic strength (Fig. S9†), from 470 nm (pure water) to 439 nm ([NaCl] = 1 M). In the excited state, a similar stabilization mechanism is less effective as some charge density is displaced to partially screen the net positive charge of the pyridinium block, thus lowering the molecular dipole (*e.g.* from 18.1 D to 10.8 D in water).

In contrast to absorption, the PL spectrum (Fig. 2A) remains almost unaltered upon increasing solvent polarity (peaking at ∼670 nm), while the PLQY is strongly reduced. This result is attributed to the complex interplay between two opposite effects: (i) a relatively higher stabilization of the ground state compared to the excited state in polar solvents, which leads to an increased S_0_–S_1_ transition energy (blue-shift); (ii) solvent cage reorganization that lowers the excited state and produces a red-shift of the transition. However, despite similar transition energies in a polar solvent, the interaction of the molecule with the environment is larger, leading to more efficient non-radiative decay. Accordingly, in lipid bilayers, which are known to have a low polarity (*ε* ∼ 4), we observe a ∼1000-fold increase in the PLQY compared to the polar water environment. This favourable feature allows mapping of the BV-1 membrane localization easily *via* common fluorescence microscopies. Both the hypsochromic solvatochromism trend in absorption and the decrease in the PLQY can be rationalized by the simulation reported in Fig. 2B, which was computed using time-dependent density functional theory (TDDFT) calculations.

### Time-resolved spectroscopy

We conducted transient absorption (TA) and time-resolved photoluminescence (TRPL) experiments to study BV-1 excitation/de-excitation dynamics in different media, namely water, ethanol, dichloromethane, and sodium dodecyl sulphate (SDS) micelle suspension. The first three solvents allowed us to explore how the behaviour of the molecule, particularly its donor–acceptor nature, is influenced by polarity, while SDS micelles were chosen to mimic the biological membrane environment. At this stage, SDS micelles were preferred over liposomes, as they produce – due to their characteristic dimensions – a relatively high scattering signal, which is not optimal for transmission measurements.

**Fig. 3:**
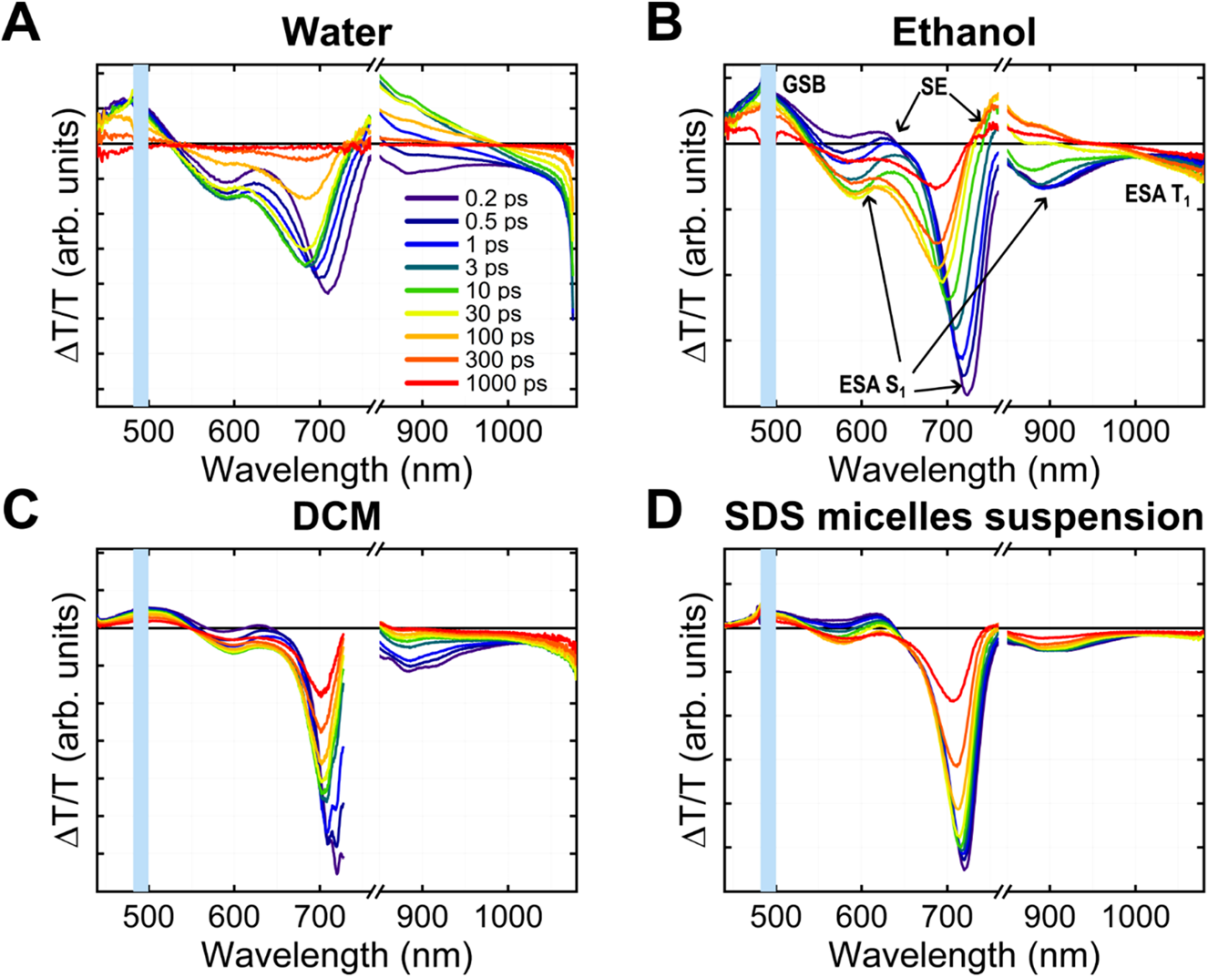
Transient absorption spectra of BV-1 in water (A), ethanol (B), dichloromethane (C) and SDS micelle suspension [100 mM] (D). The system was excited at 490 nm and probed with two different white light super-continuums to probe both the visible and the NIR range. The solutions were flowed using a peristaltic pump, to avoid photobleaching of the samples.

The TA spectra (Fig. 3A–D) show different convoluted features. First, the positive peak between 450 and 550 nm, which overlaps with the main absorption band of BV-1, can be attributed to the ground state bleaching (GSB). Additionally, we observed two other positive bands peaking around 630 nm and 750 nm, which are related to stimulated emission (SE). Interestingly, the higher energy SE band appears immediately upon excitation, while the lower energy band emerged afterwards. This suggests the presence of two separate emissive states of BV-1, both contributing to the steady-state PL spectra shown in Fig. 2A. This mechanism is further supported by TRPL data (Fig. S13†), which reveals the existence of two fluorescent states of BV-1: the first one is populated as soon as the photoexcitation occurs, whilst the other, which lies at lower energy, is populated at a slower timescale (∼10 ps in water, ∼100 ps in DCM). It is worth noting that the rise time is far too long to be due to intraband vibrational relaxation, and is most likely due to conformational relaxation of the molecule as suggested by TDDFT results (see Fig. S10†).^26^

Conversely, the negative transient bands located at 580, 700, and 900 nm and above 1000 nm are associated with excited state absorption (ESA) from various optical transitions. With the support of TDDFT calculations, we related the first three transitions to the absorption from the first singlet excited state S_1_ to higher singlet states (S_1_ → S_*n*_, additional details are reported in Fig. S10†). The transition at lower energy is likely related to triplet state absorption. The blue shift (10–20 nm) observed in the singlet state ESA bands at longer probe delays provided further evidence of the conformational relaxation of S_1_, which lowers the energy of the state and increases the transition energy. This relaxation process was also reproduced through TDDFT calculations. The computed ESA spectra for both the singlet S_1_ and triplet T_1_ states are reported in Fig. S12.†

We recognized nearly identical features across different media, with slight shifts in wavelengths and dynamics. The conformational relaxation seems to occur faster in water and becomes slower in ethanol and DCM. Moreover, we did not observe relaxation features in the SDS micelle suspension, likely due to conformational constraints experienced by the molecule when partitioned within the micelles. Furthermore, we carried out TA experiments also in an *E. coli* cell suspension (Fig. S15†), revealing that both spectra and dynamics are substantially identical to those observed in SDS micelles, despite the relatively high scattering noise in cells. Notably, this confirmation supports the suitability of SDS micelles as appropriate models for spectroscopy experiments,^27,28^ thus indicating that the BV-1 excitation landscape observed in micelles can be reliably translated to *E. coli*, thereby facilitating the rationalization of the PDI mechanism in cells.

### Summary of BV-1 photophysics

**Fig. 4:**
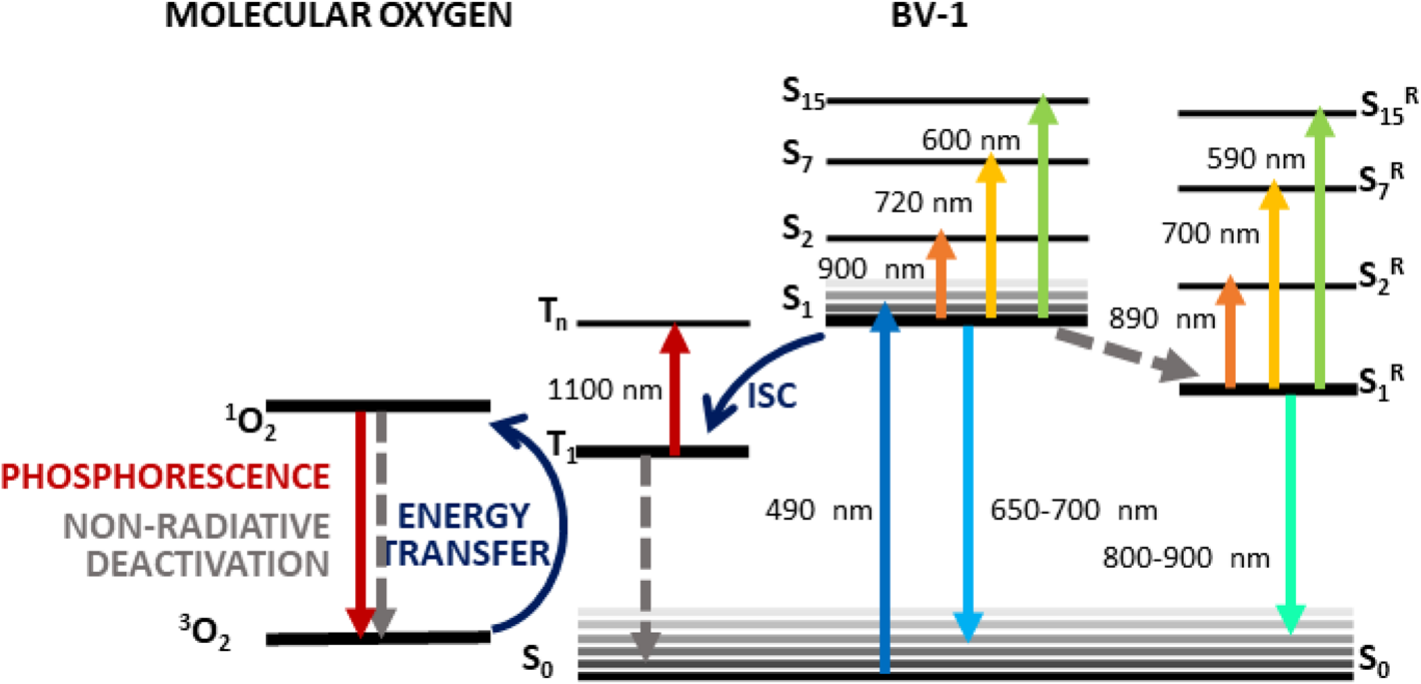
Schematic of the photophysics of BV-1 as suggested by the TA data together with calculations.

The optical spectroscopy data provide insights into the photophysical characteristics of BV-1, which are summarized in the sketch in Fig. 4. Upon excitation at 490 nm, the molecule reaches the first singlet excited state S_1_, which is recognized by the three ESA transitions observed at 600, 720 and 900 nm. As revealed by the calculations (Fig. S10†), these ESA bands correspond to the transitions S_1_ → S_15_, S_1_ → S_7_, and S_1_ → S_2_, respectively, all having high oscillator strength. From the S_1_ state, BV-1 undergoes three competitive deactivation pathways: (i) decay to the ground state, associated with fluorescence around 600–650 nm which is observed as the stimulated emission signal in the TA spectra; (ii) conformational relaxation to a long-living state, S_1_^R^, associated with the blue-shifted ESA bands (at 590, 700, and 890 nm) and emission in the NIR range; (iii) ISC to the triplet manifold, leading to an ESA band above 1000 nm. These pathways highlight the intricate photophysical behaviour of BV-1 and provide a comprehensive understanding of its excited state dynamics.

As pointed out before, the behaviour of BV-1 is strongly affected by the polarity of the solvent, owing to its di-cationic nature and distinct donor–acceptor structure. Indeed, in polar environments, such as water, the relaxation process of the S_1_ state occurs more rapidly due to solute–solvent interactions which permit dissipation paths responsible for the more efficient radiationless deactivation resulting in a lower PLQY. Consequently, relaxation into S_1_^R^ is favoured in polar solvents, whereas ISC towards the triplet state becomes more predominant in low-polarity environments. Consistently, in apolar SDS micelle suspensions and *E. coli* cells, we did not observe any signs of the relaxation process to S_1_^R^. This prompted us to verify whether BV-1 is capable of transferring energy to molecular oxygen, leading to the production of singlet oxygen for potential applications in the photodynamic inactivation of bacteria. To asses this, we exploited the reaction of 1,3-diphenylisobenzofuran (DPBF) with singlet oxygen, resulting in the formation of an endoperoxide and the subsequent disappearance of its characteristic absorption band at 415 nm.^29^ We put under illumination a solution of DPBF (50 μM) and BV-1 (5 μM) with a 470 nm LED for 20 minutes and monitored the absorption spectrum of the solution (Fig. S14†). The more rapid decomposition of DPBF in the presence of BV-1 indicates that the molecule indeed functions as a photosensitizer for singlet oxygen, demonstrating that the triplet state of BV-1 can transfer its excitation energy to molecular oxygen. By fitting the dynamic traces, we determined the decay rates *k*_DPBF_ = 0.21 min^−1^ and *k*_DPBF+BV1_ = 0.32 min^−1^, in case of pure DPBF and DPBF together with BV-1, respectively. Thus, we were able to estimate the BV-1 singlet oxygen production rate, which is 
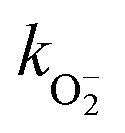
 = 0.11 min^−1^.

### Photodynamic antibacterial activity

**Fig. 5:**
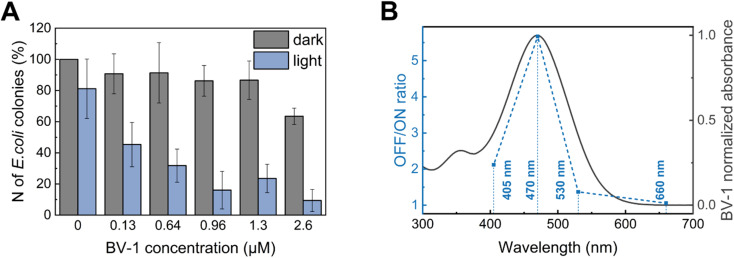
(A) Histogram representing the biocidal activity of BV-1 against *E. coli* under dark and light conditions. Illumination/dark cycles were conducted by exposing the colonies to 470 nm light for 10 minutes, followed by 10 minutes under dark conditions (3 times). (B) *E. coli* cultures were loaded with 0.5 μg mL^−1^BV-1 and exposed to 470 nm light at different wavelengths (same illumination protocol). The action curve is obtained by computing the ratio of the number of colonies grown in the dark to the number of colonies grown under illumination. All conditions and controls were measured in triplicate.

Finally, we evaluated the minimum inhibitory concentration (MIC) of BV-1 against *E. coli* under both dark conditions and light (470 nm) (Fig. 5A). The data reveal that illumination at 470 nm does not significantly affect the number of colony forming units (CFU) in the BV-1-free sample. However, upon illumination the addition of 0.12 μM BV-1 leads to a substantial decrease of CFU by 50%. Further increasing the BV-1 concentration up to 0.64 μM and 0.96 μM causes a CFU reduction of 65% and 82%, respectively. Conversely, under dark conditions BV-1 does not exhibit any significant toxicity up to 1.3 μM, with a 30% decrease in CFU at 2.6 μM. These findings indicate that illumination enhances the antibacterial activity of BV-1, as the MIC decreases from 2.6 μM to 0.12 μM. Additionally, we assessed mammalian cell viability (HEK-293T cells) upon exposure to BV-1 and light using the well-established AlamarBlue viability assay (Fig. S16†). In this case, we observed a phototoxic effect at a concentration of 5 μM, a value which is more than 30 times higher than the MIC in *E. coli* cells.

To test whether this strong light-activated antibacterial action is related to the excited state pathways of BV-1, we recorded a “BV-1 action curve” (Fig. 5B). This experiment was conducted by calculating the ratio of the CFU in the dark to the CFU under illumination at a fixed concentration of BV-1 (0.64 μM) as a function of the excitation wavelength (OFF/ON ratio). Remarkably, we observed an almost perfect overlap of the action spectrum with the absorption spectrum of BV-1, providing further confirmation that the photodynamic antibacterial activity is solely attributed to the photoactivation of BV-1.

## Conclusions

In this work, we designed and thoroughly characterised a new photosensitizer, namely BV-1, which exhibits some intriguing features for the effective eradication of bacterial pathogens. First, BV-1 possesses a strong ability to associate with bacterial cells and readily partitions into their plasma membrane, which serves as the bio-target for reactive oxygen species to induce effective photodynamic inactivation (PDI). In addition, its localization at the membrane level is accompanied by a strong fluorescence turn-on, making BV-1 suitable for imaging-guided PDI and other surface disinfection applications. Through detailed optical spectroscopy measurements, corroborated by calculations, we determined that BV-1 behaves as an intramolecular donor–acceptor chromophore upon light excitation, exhibiting strong solvatochromic properties. Specifically, we observed that in polar environments the deactivation from the lowest-energy singlet state, which possesses a charge-transfer character, occurs preferentially through conformational relaxation and rapid deactivation. Conversely, in the apolar solvents and within the membrane environment, BV-1 prefers to undergo intersystem-crossing towards a triplet state. In turn, the triplet state can efficiently transfer energy to molecular oxygen, making BV-1 a suitable intramembrane photosensitizer. Indeed, following illumination with visible light, BV-1 effectively inhibits the *E. coli* proliferation at a low minimum inhibitory concentration (0.12 μM), which is below state-of-the-art values.^30,31^ For this reason, this study provides a novel strategy for the development of innovative tools to combat infections caused by multidrug-resistant bacteria.

## Materials and methods

### BV-1 synthesis

Reagents and solvents were bought from TCI, BLDpharm, and Sigma-Aldrich, and used as received. Chromatographic purifications were performed using Davisil LC 60A silica gel (pore size 60 Å, 70−200 μm). NMR spectra were collected on a Bruker NMR Avance 400 NEO.

#### Synthesis of intermediate 1

In a microwave test tube, 5-bromo-2-thiophencarboxaldehyde (1.48 g, 7.75 mmol) and 4-picoline (1.07 g, 11.3 mmol) are dissolved in a mixture of acetic acid (2.8 mL) and acetic anhydride (1.2 mL). The solution is heated by microwave treatment for 6 hours at 140 °C, and then allowed to cool down to room temperature. 4.0 mL of deionized water are added, followed by 4.36 g of NaOH. The mixture is then extracted with DCM (3 × 15 mL). The organic phase is dried over Na_2_SO_4_, then filtered and concentrated under reduced pressure. The solution is then passed over a silica plug, eluting with DCM (to remove unreacted 5-bromo-2-thiophencarboxaldehyde), and subsequently with AcOEt to recover the product. After evaporation of the solvent under reduced pressure, 1.04 g of product 1 are recovered as a brown powder (3.91 mmol, 50% yield).

#### Synthesis of intermediate 2

In a 50 mL roundbottom flask, 1 (1.000 g, 3.757 mmol), 2-(5′-hexyl-[2,2′]bithiophenyl-5-yl)-4,4,5,5-tetramethyl-[1,3,2]dioxaborolane (1.505 g, 3.999 mmol), and Pd(dtbpf)Cl_2_ (52.0 mg, 0.0798 mmol) are dispersed in 7.5 mL of 2 wt% Kolliphor EL/toluene 9 : 1 emulsion. The mixture is stirred at room temperature for 5 minutes, then warmed at 60 °C. Et_3_N (810 mg, 8.00 mmol) is then added, and the reaction is stirred for two hours and then warmed to room temperature. 50 mL of AcOEt are added, and the mixture is extracted. The organic phase is dried over Na_2_SO_4_, then filtered and concentrated under reduced pressure. The solution is then passed over a silica plug, eluted with AcOEt. After evaporation of the solvent under reduced pressure, the crude is crystallized from iPrOH to afford 1.360 g of product 2 (3.122 mmol, 83% yield) as a brownish-orange powder.

#### Synthesis of intermediate 3

In a 50 mL roundbottom flask, 2 (560 mg, 1.29 mmol) is dissolved in 20 mL of refluxing acetonitrile. When 2 is completely dissolved, 1,6-dibromohexane (2.203 g, 9.030 mmol) is added, and the reflux continued for 6 hours. A deep red precipitate forms, which is filtered off and washed with toluene. The powder is dried in a vacuum until weight stabilization. 800 mg (1.18 mmol), 91% yield.

#### Synthesis of BV-1

In a 50 mL roundbottom flask, 3 (600 mg, 0.883 mmol) and 2-dimethylaminoethanol (886 mg, 9.94 mmol) are refluxed in 10 mL of acetonitrile. After 9 hours, the red precipitate is filtered off and dried in a vacuum until weight stabilization. 503 mg (0.653 mmol), 74% yield. Anal. calcd for C_35_H_48_Br_2_N_2_OS_3_: C, 54.68; H, 6.29; N, 3.64. Found: C, 54.70; H, 6.15; N, 3.41.

### Quantum chemical calculations

The most stable configurations of BV-1 in solution have been found using a conformer–rotamer search algorithm based on the GFN2-xTB tight-binding Hamiltonian.^32–34^ Lowest-energy configurations have been used as starting points for (time-dependent) density functional theory studies, carried out using the ORCA suite of programs.^35,36^ All simulations have been performed using the revised PBE0 functional together with the def2-TZVPP basis set. Further details of computational methods are provided in the ESI.

### Chemicals

All chemicals (dimethyl sulfoxide, acetonitrile, ethanol, dichloromethane, chloroform, methanol and sodium dodecyl sulphate (SDS)) were purchased from Sigma-Aldrich. BV-1 solutions were prepared in the different organic solvents to reach a concentration of 25 μM, unless otherwise specified. SDS micelle suspension in Milli-Q water was obtained by suspending SDS above its critical micellar concentration (100 mM). POPC was purchased from Avanti Polar Lipids.

### POPC liposomes

Liposomes were prepared through a standard extrusion procedure. 1-Palmitoyl-2-oleoyl-*sn*-glycero-3-phosphocholine (POPC) in chloroform solution mixed with methanol (1 : 1) was dried out under high vacuum with the use of a rotary evaporator, and the resulting thin lipid film was kept overnight at −20 °C. The film was then hydrated with a buffer solution (10 mM Tris, 100 mM NaCl, pH 7.4) and subjected to seven freeze–thaw cycles and extrusions through a polycarbonate membrane (pore size 100 nm) at room temperature. The samples were kept at 4 °C, at a liposome concentration of 5 mg mL^−1^ and diluted at 1 mg mL^−1^ for the experiments. The vesicles were used within the week of preparation.

### Steady-state UV-vis and PL measurements

UV-vis absorption measurements were performed employing a Perkin ElmerLambda 1050 spectrophotometer, with deuterium (180–320 nm) and tungsten (320–3300 nm) lamps, a monochromator and three detectors (photomultiplier 180–860 nm, InGaAs 860–1300 nm, and PbS 1300–3300 nm). Absorption spectra were normalized according to a reference spectrum taken at 100% transmission (without the sample), 0% transmission (with an internal shutter), and in the presence of the reference solvent. For the PL measurements, an iHR320Horiba NanoLog fluorometer was used, equipped with a xenon lamp, two monochromators, and two detectors (photomultiplier and InGaAs). Samples were excited at 480 nm.

### Transient absorption measurement

For the femtosecond TA measurements, we used an 800 nm Ti : Al_2_O_3_ laser with a repetition rate of 1 kHz and a pulse width of 100–150 fs. We excited the solutions with a 490 nm pump beam that was generated by means of a nonlinear optical parameter amplifier. For probing in the visible range, we generated a white light beam with a 2 mm Ti : Al_2_O_3_ plate filtered with a BG39, while probing in the NIR was done by generating white light exploiting a 4 mm YAG crystal, together with an LP850 filter to remove the laser fundamental wavelength.

The excitation energy density was about 1.7 μJ mm^−2^. To avoid photobleaching, the solutions were circulated by means of a peristaltic pump (∼30 rpm).

### *E. coli* cultures

Experiments were conducted using *Escherichia coli* (*E. coli*) strain ATCC25922. For bacterial growth, a single colony was inoculated in Luria–Bertani (LB) broth and incubated overnight at 37 °C with shaking at 215 rpm, until the stationary phase was reached. Then, the bacterial suspension turbidity (expressed as optical density at 600 nm; OD_600_) was diluted to OD_600_ = 1 in LB broth, without antibiotics. Bacteria were then centrifuged, and the obtained pellet was resuspended in a phosphate-buffered saline (PBS) aqueous solution.

### Confocal imaging

For confocal imaging, the *E. coli* culture was incubated at a concentration of 5 μM (from a 2 mM stock solution) in the PBS samples, prepared as described above. BV-1 was incubated for 1 h at 37 °C. After incubation, bacteria were centrifuged, and the obtained pellet was resuspended in PBS solution in order to remove cell unbound molecules. 2 μL of the *E. coli* suspension was then dropped on a ∼1 mm LB pad and covered with a glass microscope slide, to reduce bacteria movement during image acquisition. The glass slide was then mounted on an inverted confocal laser scanning microscope (Nikon Eclipse Ti2, Nikon Instruments). Acquisitions were performed using an Olympus 60× oil objective.

### Cell association experiments

*E. coli* cells suspended in PBS were stained with different concentrations of BV-1 (50, 10 and 0 μM) and kept at 37 °C for 60 minutes in the dark. The samples were then centrifuged and 200 μL of each supernatant was transferred to a clean 96-well plate for UV-vis absorption with a Tecan Spark10M plate reader. Absorbance was measured at 480 nm. Control samples with no cells were treated the same, and their absorbance values represented the complete molecule for reference. All conditions and controls were measured in triplicate.

### MIC

*E. coli* cultures in LB broth (OD_600_ = 0.005) were loaded with different concentrations of BV-1 (2.6, 1.3, 0.96, 0.64, 0.13 and 0 μM). Half of the samples were exposed to light (LED 470 nm) using the following illumination protocol: 10 minutes of light followed by 10 minutes under dark conditions, repeated three times (light power density 14 mW cm^−2^). The samples were then seeded on LB agar plates and kept in the dark at 37 °C overnight. The number of colonies grown under the different conditions was then counted and normalized to the control sample (grown in the dark and not loaded with BV-1). All conditions and controls were measured in triplicate.

### Action curve

*E. coli* cultures in LB broth (OD_600_ = 0.005) were loaded with 0.64 μM BV-1. Half of the samples were exposed to light (LED at 405, 470, 530 or 660 nm) using the following illumination protocol: 10 minutes of light followed by 10 minutes under dark conditions, repeated three times. The samples were then seeded on LB agar plates and kept in the dark at 37 °C overnight. The number of colonies grown under the different conditions was then counted. The action curve is obtained by computing the ratio of the number of colonies grown in the dark to the number of colonies grown under illumination. All conditions and controls were measured in triplicate.

### AlamarBlue viability assay on HEK-293T cells

HEK and C2C12 cells were cultured in T-25 flasks containing Dulbecco's modified Eagle's medium (DMEM) supplemented with fetal bovine serum (10%), glutamine (2 mM), penicillin (100 IU mL^−1^) and streptomycin (100 μg mL^−1^). Culture flasks were maintained in a humidified incubator at 37 °C with 5% CO_2_ and, when at confluence, plated at a density of 3000 cells per cm^2^. After being exposed to BV-1 for 5 minutes at concentrations of 0, 1, 5 and 10 μM, some samples were exposed to light for 30 seconds (470 nm, 300 μW mm^−2^). Cell proliferation was checked with a standard AlamarBlue assay performed 0, 48 and 96 hours after exposure to BV-1. Briefly, the AlamarBlue reagent (Invitrogen) was diluted 11 : 1 with DMEM without phenol red. 1 mL of the obtained solution was added to each well. The solution was incubated for at least 3 h in the incubator. Once removed from the well, the emission at 590 nm of three aliquots per well was measured. The emission value was acquired 3 times per aliquot to obtain a reliable measurement. All conditions and controls were measured in triplicate.

## Data availability

The datasets supporting this article have been uploaded as part of the ESI.†

## Author contributions

A. M. carried out all the spectroscopy experiments, contributed to data interpretation and wrote the first draft of the manuscript. S. M., L. B and A. Z. performed the synthesis of BV-1 and contributed to manuscript writing. G. M. performed the calculations and contributed to spectroscopy data interpretation and manuscript writing. M. M., together with A. M., carried out the biological experiments. G. M. P. and G. L. supervised the experiments and contributed to data interpretation and manuscript writing.

## Conflicts of interest

The authors declare no competing financial interest.

## Supplementary Material

SC-014-D3SC01168B-s001
