# Supplementary material for: A membrane intercalating metal-free conjugated organic photosensitizer for bacterial photodynamic inactivation
Source: Chem Sci. 2023 Jul 5;14(30):8196–205. doi: 10.1039/d3sc01168b (PMC10395270; doi:10.1039/d3sc01168b)
Supplement: SC-014-D3SC01168B-s001 [file SC-014-D3SC01168B-s001.pdf]

Electronic Supplementary Material (ESI) for Chemical Science.
This journal is © The Royal Society of Chemistry 2023

Membrane Intercalating Metal-free Conjugated Organic Photosensitizer for Bacterial Photodynamic Inactivation

Arianna Magni[1,2], Sara Mattiello[3], Luca Beverina[3], Giuseppe Mattioli[4], Matteo Moschetta[2], Anita Zucchi[3], Giuseppe Maria Paternò[1,2*] and Guglielmo Lanzani[1,2*]

[1]Department of Physics, Politecnico di Milano, 20133 Milan, Italy

[2]Center for Nano Science and Technology @PoliMi, Istituto Italiano di Tecnologia, 20134 Milan, Italy

[3]Department of Materials Science, University of Milano-Bicocca, 20125 Milan, Italy

[4]CNR - Istituto di Struttura della Materia, I-00015 Monterotondo Scalo, Italy

[*]corresponding authors: giuseppemaria.paterno@polimi.it ; guglielmo.lanzani@iit.it

Supplementary materials

Synthesis details of BV–1

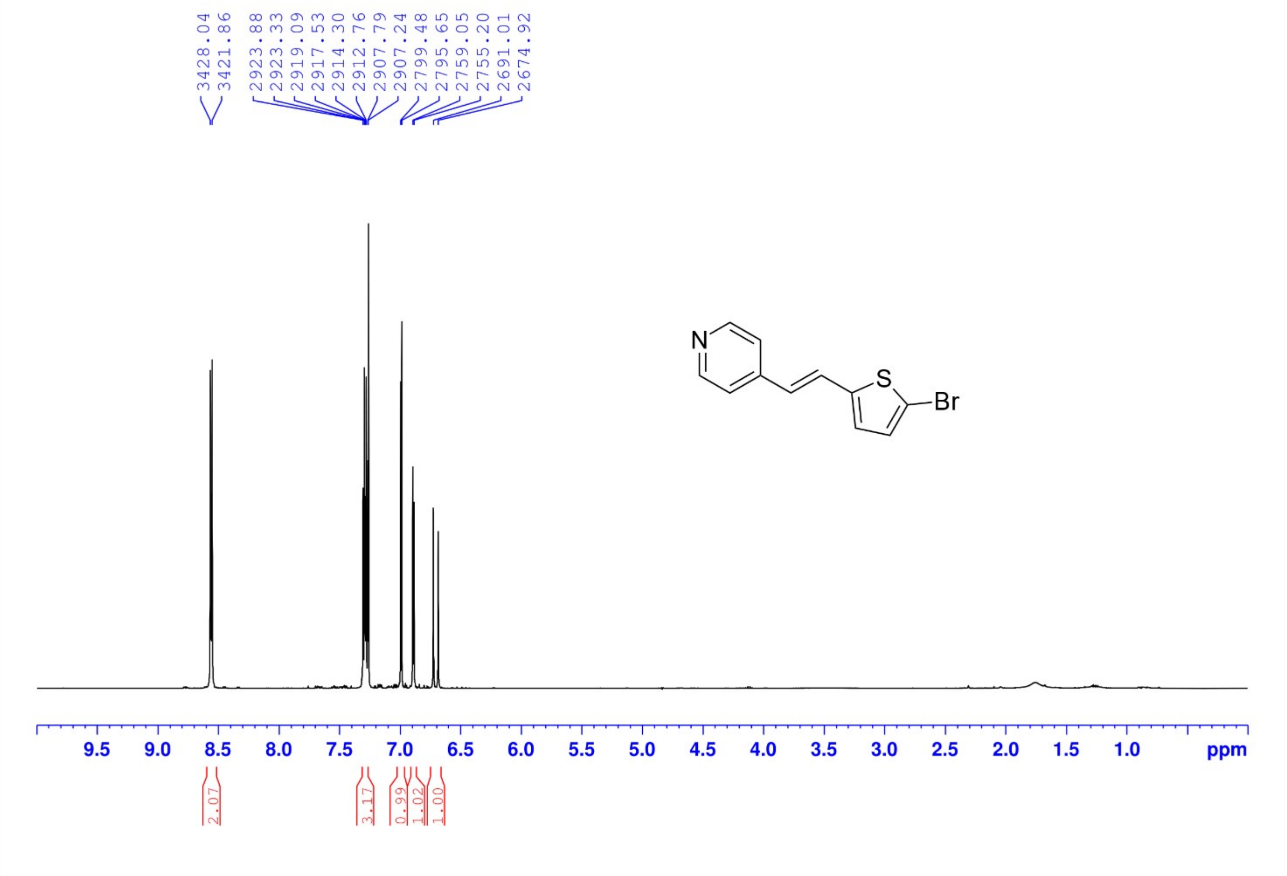

Figura S1

^{1}H NMR in $CDCl_3$ of derivative **1**.

H NMR ($CDCl_3$, 400 MHz): δ 8.56 (d, J=6.2 Hz, 2H), 7.31-7.27 (m, 3H), 6.99 (d, J=3.8 Hz, 1H), 6.89 (d, J=3.8 Hz, 1H), 6.71 (d, J=16.1 Hz, 1H) ppm

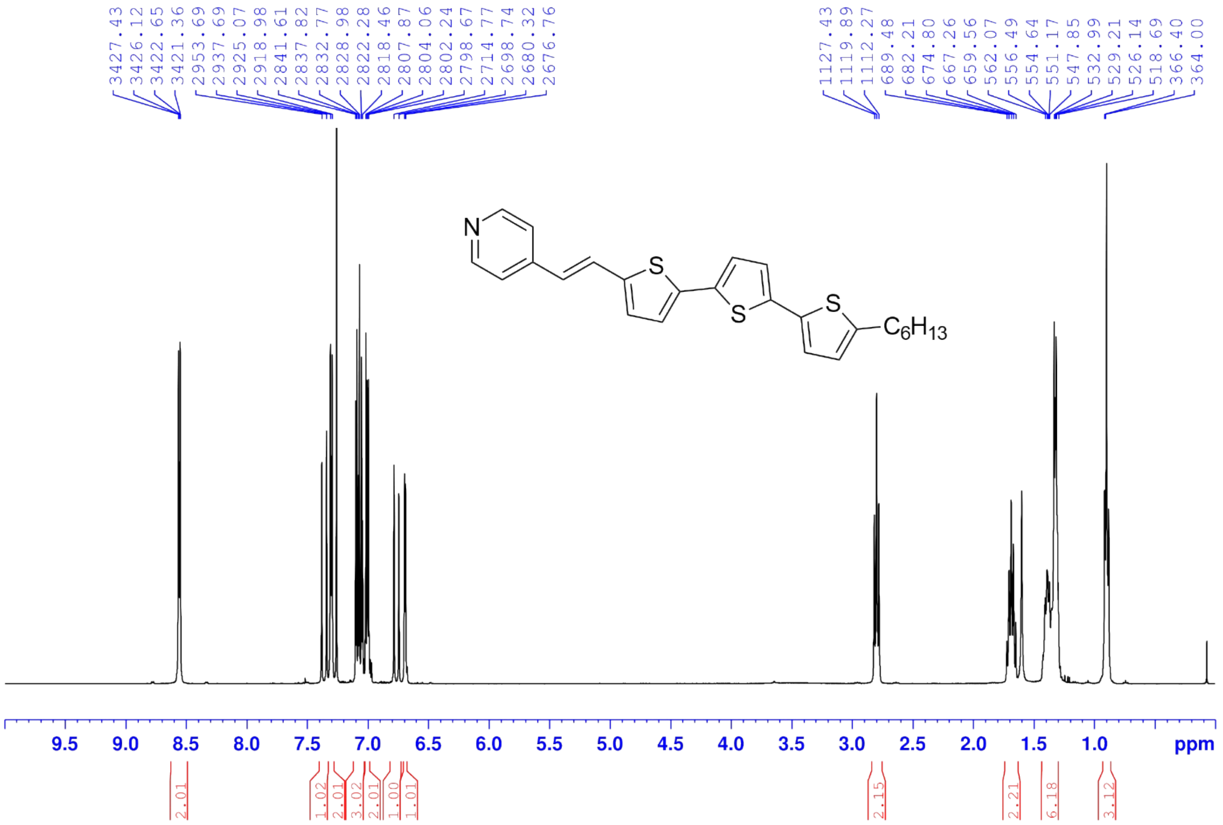

Figure S2

^{1}H NMR in $CDCl_3$ of derivative **2**.

^{1}H NMR ($CDCl_3$, 400 MHz): δ 8.56 (d, J=5.7 Hz, 2H), 7.36 (d, J=16.0 Hz, 1H), 7.31 (d, J=6.0 Hz, 2H), 7.10 (d, J=3.8 Hz, 1H), 7.08 (d, J=3.8 Hz, 1H), 7.05 (d, J=3.8 Hz, 1H), 7.02-7.00 (m, 2H), 6.77 (d, J=6.0 Hz, 2H), 6.69 (d, J=3.5 Hz, 1H), 2.80 (t, J=7.6 Hz, 2H), 1.69 (m, 2H), 1.43-1.27 (m, 6H), 0.89 (t, J=7.0 Hz, 3H) ppm

^{13}C NMR in $CDCl_3$ of derivative **2**.

^{13}C{^{1}H} NMR ($CDCl_3$, 100 MHz): δ 150.22, 146.01, 144.18, 140.23, 137.83, 137.61, 135.00, 134.22, 129.19, 125.84, 125.12, 124.91, 124.79, 123.89, 123.64, 120.48, 31.55, 30.20, 28.74, 22.56, 14.07 ppm.

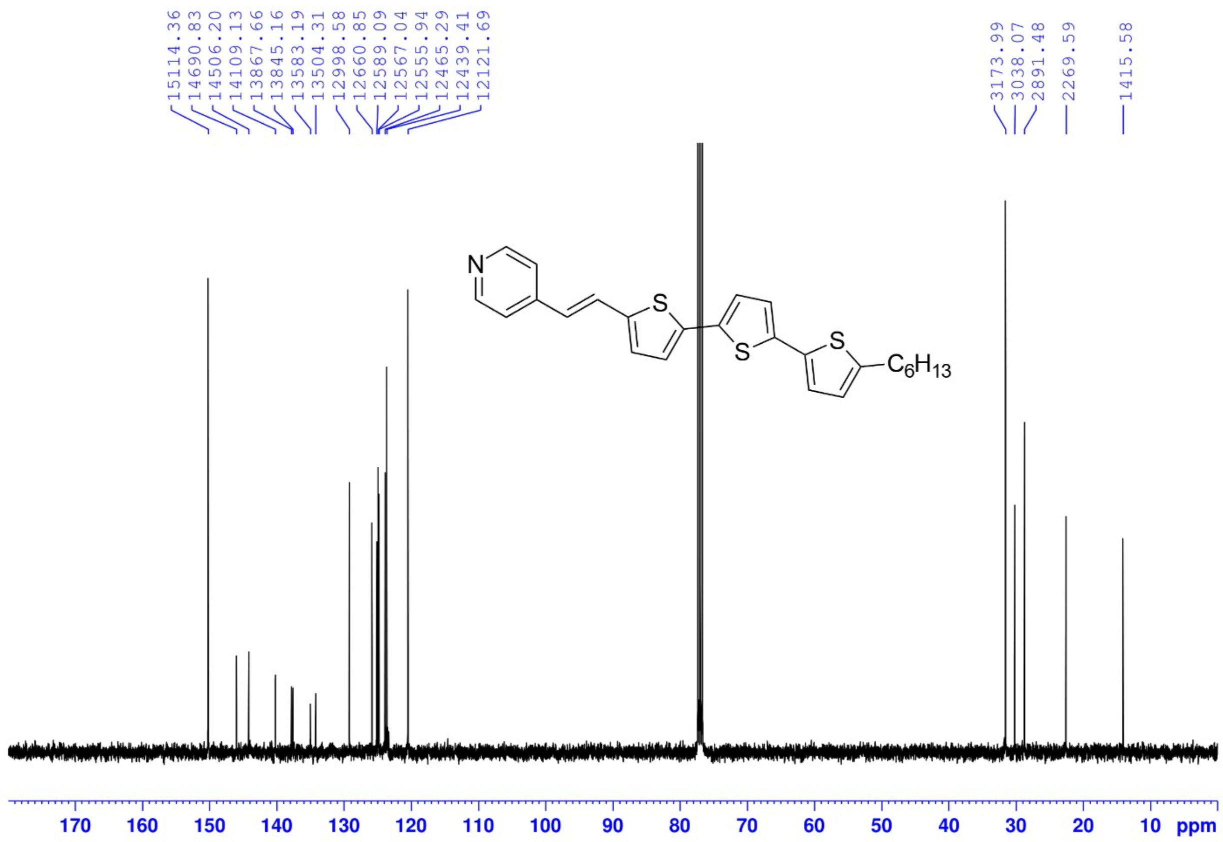

Figure S3

^{13}C NMR in $CDCl_3$ of derivative **2**.

$^{13}C\{^1H\}$ NMR ($CDCl_3$, 100 MHz): δ 150.22, 146.01, 144.18, 140.23, 137.83, 137.61, 135.00, 134.22, 129.19, 125.84, 125.12, 124.91, 124.79, 123.89, 123.64, 120.48, 31.55, 30.20, 28.74, 22.56, 14.07 ppm.

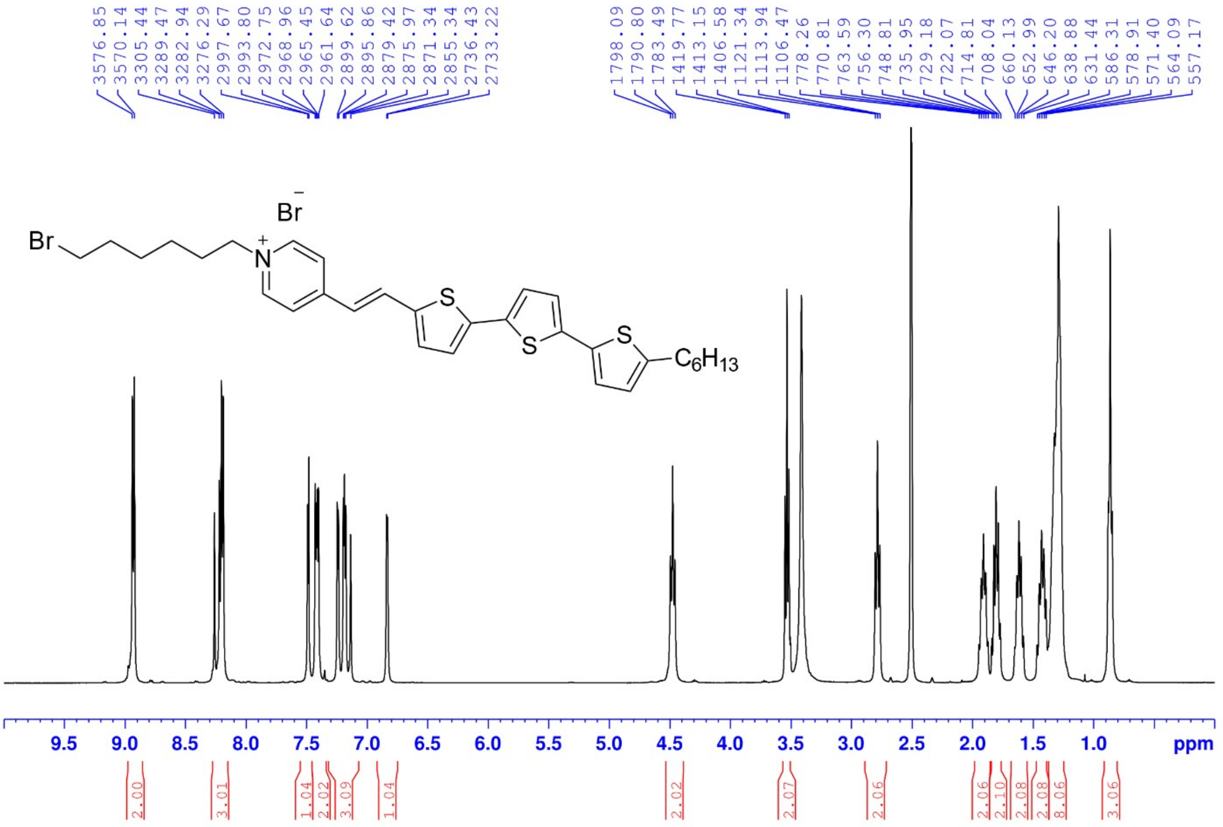

Figure S4

^{1}H NMR in DMSO-d_6 of derivative **3**.

^{1}H NMR (DMSO-d_6, 400 MHz): δ 8.92 (d, J=7.1 Hz, 2H), 8.25-8.19 (m, 3H), 7.49 (d, J=4.0 Hz, 1H), 7.44 (d, J=3.8 Hz, 1H), 7.42 (d, J=3.8 Hz, 1H), 7.26 (d, J=3.8 Hz, 1H), 7.20 (d, J=3.6 Hz, 1H), 7.17 (d, J=16.0 Hz, 1H), 6.85 (d, J=3.6 Hz, 1H), 4.47 (t, J=7.4 Hz, 2H), 3.54 (t, J=6.7 Hz, 2H), 2.80 (t, J=7.5 Hz, 2H), 1.95-1.88 (m, 2H), 1.84-1.77 (m, 2H), 1.66-1.59 (m, 2H), 1.47-1.26 (m, 10 H), 0.87 (t, J=7.0 Hz, 3H) ppm

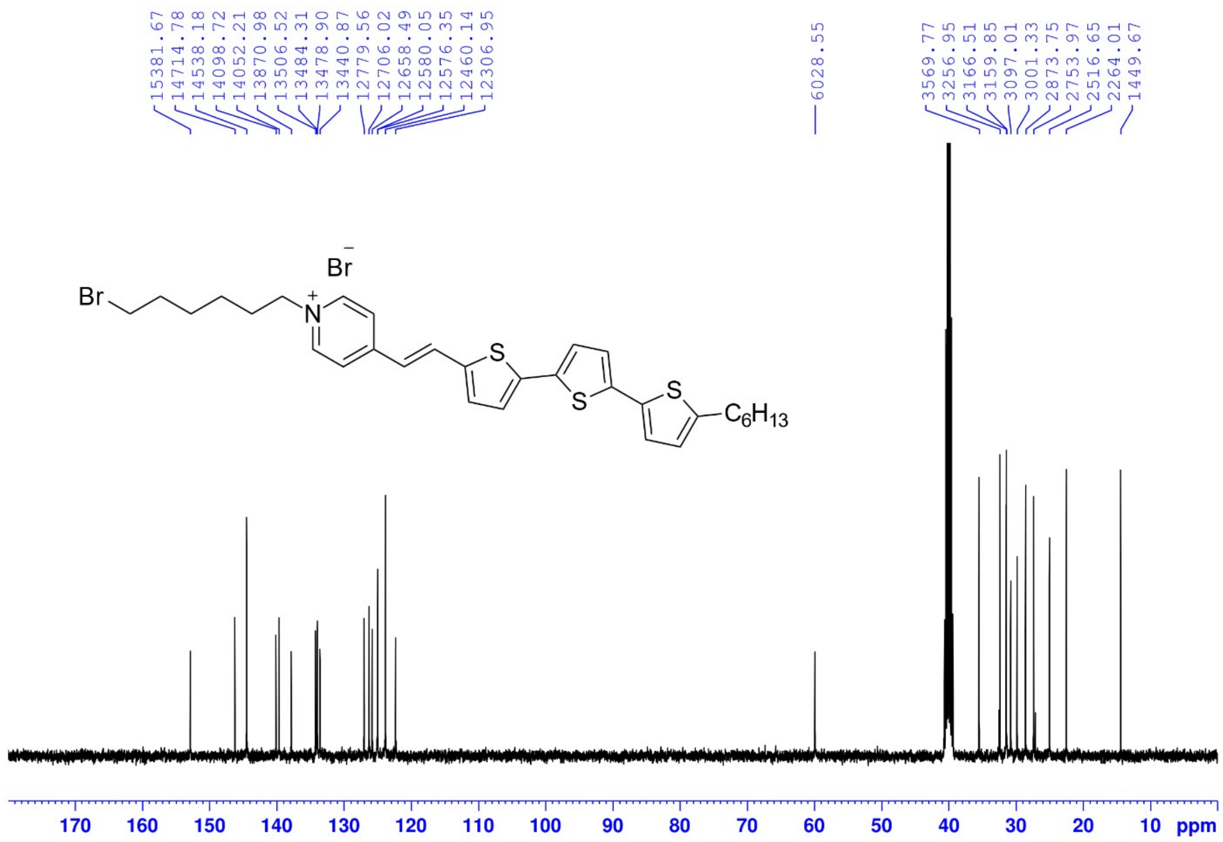

Figure S5

^{13}C NMR in DMSO-d_6 of derivative **3**.

$^{13}C\{^1H\}$ NMR (DMSO-d_6, 100 MHz): δ 152.88, 146.25, 144.50, 140.13, 139.67, 137.87, 134.24, 134.02, 133.97, 133.59, 127.02, 126.29, 125.81, 125.03, 125.00, 123.84, 122.32, 59.92, 35.48, 32.37, 31.47, 31.41, 30.78, 29.83, 28.56, 27.37, 25.01, 22.50, 14.41 ppm

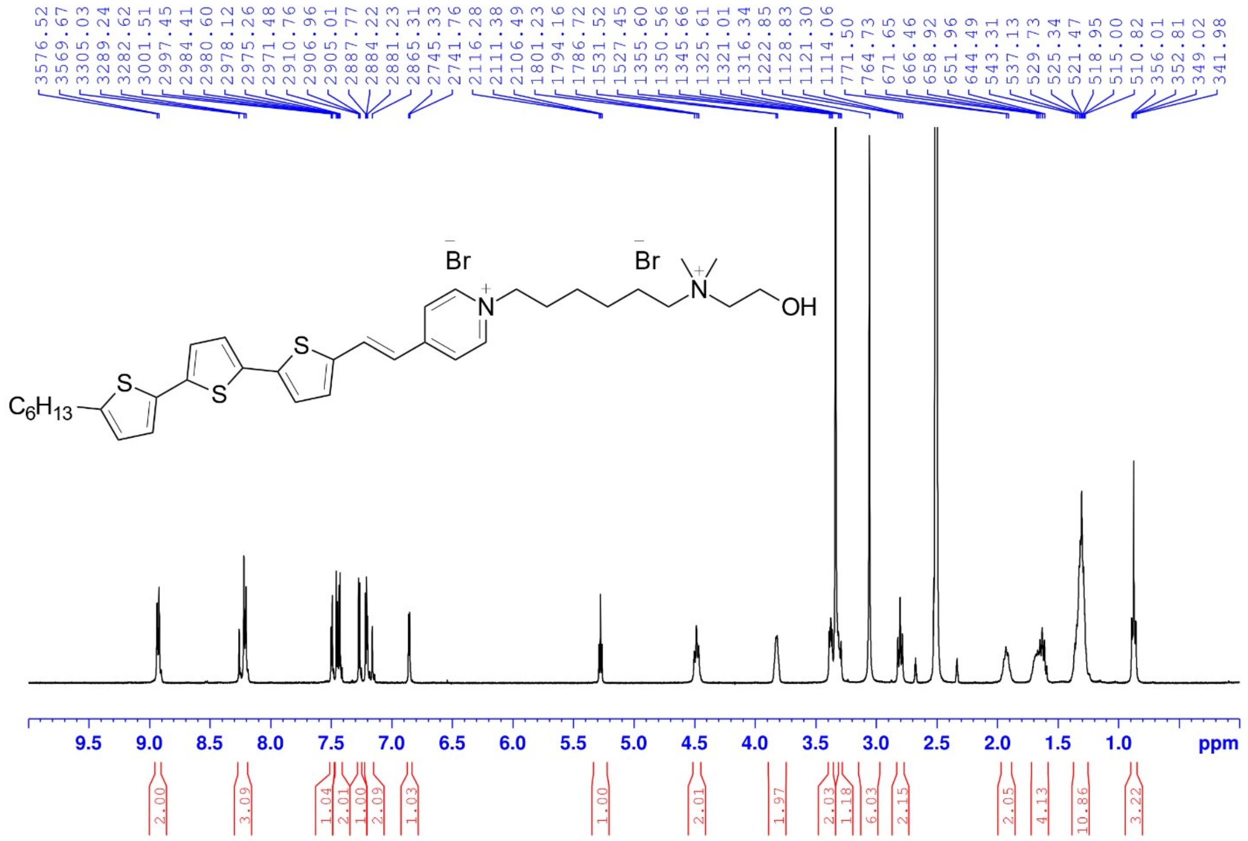

Figure S6

^{1}H NMR in DMSO-d_6 of derivative **BV–1**.

1H NMR (DMSO-d6, 400 MHz): δ 8.93 (d, J=7.1 Hz, 2H), 8.26-8.20 (m, 3H), 7.50 (d, J=4.0 Hz, 1H), 7.45 (d, J=3.8 Hz, 1H), 7.43 (d, J=3.8 Hz, 1H), 7.27 (d, J=3.8 Hz, 1H), 7.21 (d, J=3.6 Hz, 1H), 7.18 (d, J=16.0 Hz, 1H), 6.86 (d, J=3.6 Hz, 1H), 5.28 (t, J=4.9 Hz, 1H), 4.48 (t, J=7.4 Hz, 2H), 3.82 (m, 2H), 3.38 (m, 2H), 3.30 (m, 1H), 3.06 (s, 6H), 2.80 (t, J=7.5 Hz, 2H), 1.93 (m, 2H), 1.68-1.59 (m, 4H), 1.36-1.28 (m, 10H), 0.87 (t, J=7.0 Hz, 3H) ppm

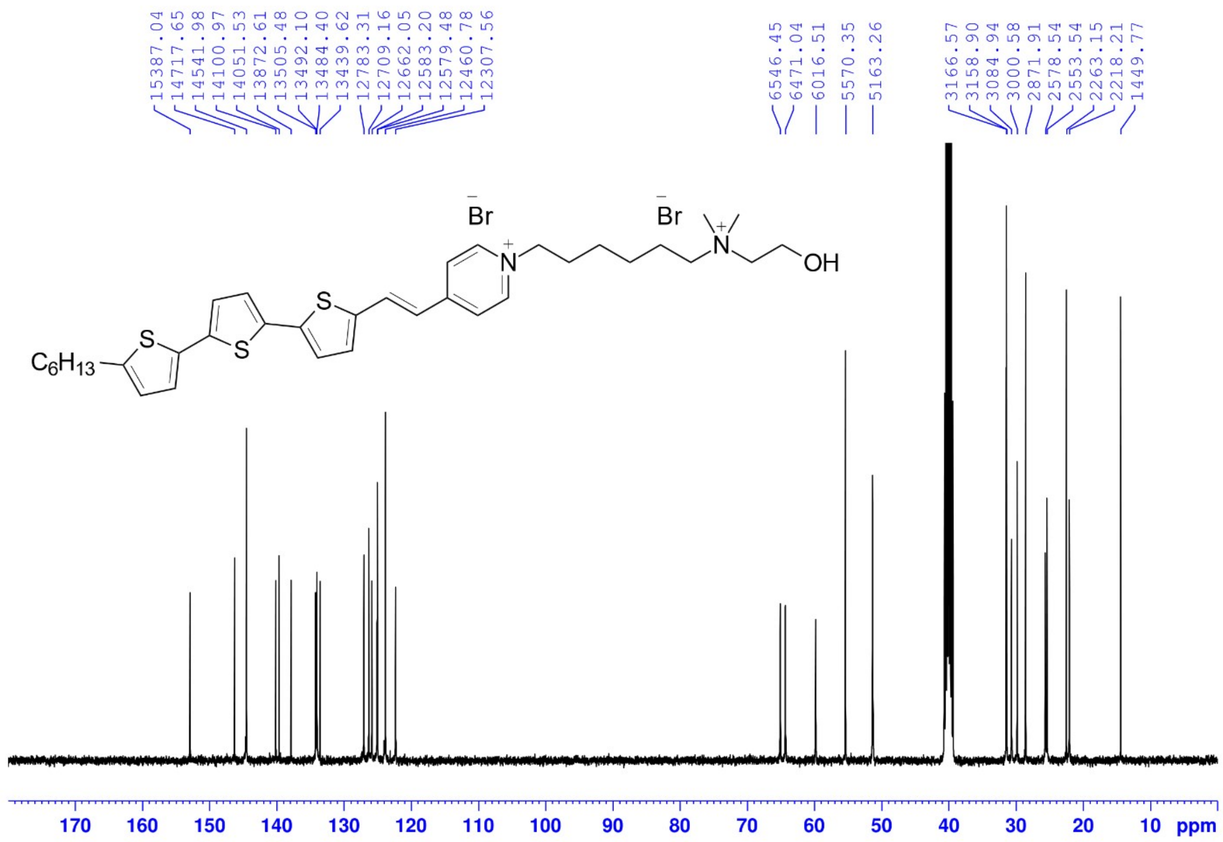

Figure S7

^{13}C NMR in DMSO-d_6 of derivative **BV–1**.

13C{1H} NMR (DMSO-d6, 100 MHz): δ 152.93, 146.28, 144.53, 140.15, 139.66, 137.88, 134.23, 134.10, 134.02, 133.58, 127.05, 126.32, 125.85, 125.07, 125.03, 123.85, 122.33, 65.07, 64.32, 59.80, 55.36, 51.32, 31.47, 31.40, 30.66, 31.41, 29.82, 28.54, 25.63, 25.38, 22.50, 22.05, 14.41 ppm

Computational methods

To ensure the full reproducibility of our computational results, we provide here a complete account of our setup. The optoelectronic properties of BV–1 have been investigated in the framework of (time dependent) density functional theory using the ORCA suite of programs.[1,2] The starting configurations of the molecule have been found using a conformer-rotamer search algorithm based on the GFN2-xTB tight-binding Hamiltonian. [3–5] A few lowest-energy conformers have been then fully optimized using a revised PBE0 hybrid functional with 37.5% of exact exchange,[6] suitable for accurately describing the strong charge-transfer excitation which dominates the absorption spectrum of BV–1**,** and the D3BJ dispersion correction.[7] The Kohn-Sham orbitals have been expanded on a Gaussian-type def2-TZVPP basis set.[8] The corresponding def2/J basis was also used as an auxiliary basis set for Coulomb fitting in a resolution-of-identity/chain-of-spheres (RIJCOSX) framework. Time-dependent DFT calculations have been performed on lowest-energy conformers using the same functional and the same basis sets; a large basis of 600 vectors connecting occupied and unoccupied Kohn-Sham orbitals has been used for the calculations of the first 30 electronic transitions of the absorption spectrum of the molecule. Solvent effects have been included in the calculations through the linear-response conductor-like polarizable continuum model (LR-CPCM).[9] Absorption and emission spectra in different solvents, including vibrational contributions, have been calculated using the excited-state dynamics (ESD) module implemented in ORCA.[10] Excited-state absorption (ESA) spectra have been calculated using the expectation value formalism, and are shown as Gaussian convolution of electronic transitions (FWHM = 4000 cm^{-1}). These transitions have been calculated both in a vertical configuration, *i.e.* in the ground state optimized geometry, as well as in the adiabatic configuration, *i.e.* in the S1 (and T1) excited-state optimized geometry. The vertical transitions can be interpreted as the limit for t=0, which correspond to the time-gated spectra taken at short probe delays before the conformational relaxation occurs. The adiabatic transitions, on the other hand, represent the limit for longer probe delays, reproducing the condition of the completely relaxed system. In all the examined solvents, we observe a blue shift of the ESA transitions going from the vertical to the adiabatic configuration that is consistent with the results of ultrafast spectroscopy measurements. Note that the computed theoretical values are shifted at higher energies with respect to the experimental values, due to computational details. The optimized geometries in Cartesian coordinates (in Å) of the BV–1 ground and S1 excited states in water are reported below.

Ground state

N 5.56292813920836 -1.78200854848512 -0.88413841318298

C	4.86771684910131	-1.01773331697871	-1.73701531972051
C	3.54138607483934	-0.76299357316084	-1.53360324854174
C	2.87610943116967	-1.29152891105466	-0.41894617822173
C	3.63971343172480	-2.08570844340536	0.44990067584582
C	4.96078146543647	-2.30696579933679	0.19462314346518
C	1.47997930117525	-1.00079075290662	-0.22976217696952
C	0.74190988319686	-1.42539836449972	0.81325642580795
C	-0.63953570156704	-1.15108969483603	1.03286490461938
C	-1.38200298517104	-1.58340648404563	2.10849796933173
C	-2.71793064052107	-1.16348952417802	2.06830977339666
C	-3.00796826289495	-0.40375007825352	0.95700502686010
S	-1.61590361032859	-0.21310702921681	-0.03589837931778
C	-4.26406154203278	0.18746398796254	0.58939818655080
S	-5.65977835207964	-0.01269737587636	1.58232185419665
C	-6.64014979114555	0.92509121348156	0.52373790046481
C	-5.90704856092266	1.36379465697571	-0.55078126280738
C	-4.56447838043292	0.94708295480825	-0.51305090111843
C	-8.03201859341480	1.15314970321627	0.81661477184632
C	-8.73835956280866	0.80534945981003	1.93622263440392
C	-10.09792571787652	1.19089952117477	1.87468137995620
C	-10.42916894544092	1.83081074071285	0.71759485971472
S	-9.05996420755950	1.95612494675755	-0.31232794137040
C	-11.76011495231101	2.35063577665750	0.29474789083867
C	-12.30913058524204	1.67141535478918	-0.95745163693719
C	-12.49936049618251	0.17584654260008	-0.79269379429240
C	-13.05820016466349	-0.49164079982993	-2.03535191995937
C	-13.24191119940698	-1.99069066235738	-1.87749599095748

C	-13.80428093654710	-2.65059757946594	-3.12374340968835
C	7.00118264279006	-1.98403222227447	-1.08497364974213
C	7.80733879223276	-0.91710925197431	-0.37292313226399
C	9.29579199770544	-1.11205999206320	-0.59187826023364
C	10.11945719699431	-0.05025625318606	0.11244958200677
C	11.61183175960329	-0.23854556752035	-0.10785190765501
C	12.36881234326199	0.83891881857314	0.63152562454778
N	13.86044404722722	0.79468876406152	0.49237507682525
C	14.42238296606101	1.93241737256201	1.26818144489005
C	14.39545483073412	-0.45934682148154	1.08104524657073
C	14.31191584621868	0.85191349700606	-0.93673671841871
C	13.72492487791505	1.94808296124675	-1.79792624381279
O	14.09030538555601	3.25565497819977	-1.43815731754083
H	5.41273054516862	-0.62441267848094	-2.57971225520123
H	3.01409915985630	-0.14762632610466	-2.24478066664916
H	3.21262880586369	-2.53923041309679	1.32803454227036
H	5.58078134747089	-2.90987509834997	0.83737722913347
H	1.02357103369322	-0.38964858176830	-0.99696889032139
H	1.19353283221123	-2.03242024832517	1.58665908508241
H	-0.95723996391032	-2.18628220280060	2.89595404749702
H	-3.44821677500260	-1.40578759010749	2.82379520275617
H	-6.32344706041080	1.97136821994769	-1.33850450388075
H	-3.83972115946787	1.19930196621158	-1.27128899764568
H	-8.29620904700147	0.29271964015294	2.77659984423822
H	-10.81496344963494	1.00129582712094	2.65814261563228
H	-11.69783837112955	3.42578227235588	0.12305316953202
H	-12.44851815375963	2.19939615896089	1.12566252389103

H	-11.63724314069332	1.86340900463122	-1.79634127443873
H	-13.26216312456031	2.13693696442664	-1.21172253720009
H	-13.16638496266970	-0.01015321584832	0.05270021889639
H	-11.54386255976490	-0.28470628658839	-0.53458799203524
H	-12.39175306652921	-0.29757819553067	-2.87957309442784
H	-14.01764174364996	-0.03588589878296	-2.29260350495797
H	-13.90329581423172	-2.18237449952782	-1.02995089246171
H	-12.28108408147797	-2.44330180254444	-1.62360474769769
H	-14.77867798767431	-2.23338901425527	-3.37840483730883
H	-13.92582647358918	-3.72439016557776	-2.98846920206172
H	-13.14509160135612	-2.49384734336879	-3.97770178874892
H	7.18386798923622	-1.96592914198672	-2.15518380702174
H	7.24442988848070	-2.97518594434123	-0.71357929587457
H	7.57970746858707	-0.94872280381796	0.69314770784769
H	7.50191813954846	0.06359175547550	-0.73922306335943
H	9.51215452449565	-1.09027635604806	-1.66153103247315
H	9.59100495098538	-2.09961077848874	-0.23269187565460
H	9.90425326132073	-0.07382291494302	1.18213791379211
H	9.82186156878343	0.93696667310880	-0.24521921300651
H	11.82155121520205	-0.20173116609526	-1.17532611360429
H	11.90335130592035	-1.22520063508661	0.24649424626762
H	12.17609140844023	0.77232848009672	1.69891773212542
H	12.05840794070766	1.82524529818298	0.30128744469351
H	14.01672103721220	2.85595564835570	0.88076985903460
H	15.50103810452874	1.92021857951537	1.15956031230568
H	14.14807840283302	1.79510306219176	2.30806352895091
H	14.06783149756365	-0.51926030569000	2.11285409729637

H	15.47785925548432	-0.42086648746346	1.03093023038785
H	14.03064949130134	-1.30746279851696	0.51693102689602
H	14.05969622332100	-0.10515302268602	-1.38168781499818
H	15.39546563851010	0.93077512787116	-0.90163964398414
H	12.63939024537225	1.91470814728027	-1.78928071734119
H	14.04101242223527	1.71545769687510	-2.81686765780056
H	15.03023242617764	3.36222986515308	-1.57361089635902

S1 Excited state

N	5.51623384422532	-1.91083516490582	-0.69547468176127
C	4.78218526543300	-1.17506846837559	-1.56124438466214
C	3.46661685972944	-0.92429113188444	-1.33705455272746
C	2.81484413992323	-1.41842553941613	-0.17955653228064
C	3.62473103236565	-2.18208674163611	0.70218681240269
C	4.93517110314219	-2.39933530274500	0.42242861061937
C	1.44964020773764	-1.14342211329099	0.03580392385139
C	0.70708117822302	-1.55796314539864	1.12115149889098
C	-0.64595045501427	-1.27838451223350	1.31830942553637
C	-1.42716286411743	-1.68744522998523	2.41932070938035
C	-2.73128620000392	-1.27244611272239	2.35474345931220
C	-3.02533370350707	-0.52074418951792	1.19953843583501
S	-1.60780108137433	-0.35666122161871	0.20612258961801
C	-4.24195492677938	0.04105819599921	0.82929032982474
S	-5.66286024714056	-0.12153112736503	1.81548189939901
C	-6.61763628689254	0.78768675477019	0.70012563334611
C	-5.84464836381602	1.19802498026194	-0.39382652417476
C	-4.53377268276481	0.78919623941573	-0.32726855496929

C	-7.98393092163037	1.03484262593167	0.93295334894061
C	-8.76023344982354	0.63282778814088	2.01116200670520
C	-10.08888985277527	1.05455029156915	1.91235667900064
C	-10.35174102873844	1.77950793098878	0.77292558224670
S	-8.94642098805857	1.94087227435344	-0.18834605491578
C	-11.65170516342954	2.35096442693403	0.33469186330582
C	-12.14800424345229	1.77353994400306	-0.99074231037138
C	-12.36294830206572	0.27299088453396	-0.94471059256573
C	-12.86346905823584	-0.29313894176283	-2.26026233580955
C	-13.07151660611632	-1.79687508190496	-2.22234464452778
C	-13.56923787605263	-2.35656137048767	-3.54287147332849
C	6.94620153317367	-2.09049687061179	-0.91600516924952
C	7.75492664248653	-0.97494685357005	-0.28201428610763
C	9.24118913234501	-1.15292751685693	-0.52730508403274
C	10.06188078215022	-0.04226019996412	0.10116583486593
C	11.55151062351238	-0.20878373348648	-0.15311785850184
C	12.30238707186212	0.92315525795415	0.50670912844500
N	13.78847310282625	0.91695540242896	0.31220465124951
C	14.34286940970567	2.10483910054979	1.01498676823149
C	14.38535253679454	-0.29251160527359	0.93347689899158
C	14.18382053578606	0.92382563856867	-1.13488701105484
C	13.53096713412786	1.96235174114653	-2.01994544146589
O	13.86329948011758	3.29534619658184	-1.72813871752371
H	5.30311104312341	-0.80819812111323	-2.43069034007868
H	2.91898324772711	-0.33868669685157	-2.05880884642833
H	3.22412124479311	-2.60814847103811	1.60666030178445
H	5.57546946419110	-2.97333641318664	1.07237934799681

H	0.96247611776285	-0.55259054949082	-0.72986278314086
H	1.16993019739884	-2.14511062635298	1.90195877953230
H	-1.00883451284979	-2.27250532674240	3.22275728000561
H	-3.47428034460933	-1.49128955224166	3.10560355347690
H	-6.25464903421426	1.77893886761968	-1.20464514445261
H	-3.79161875225416	1.00999548303470	-1.07762775918436
H	-8.36754882573225	0.05271830559577	2.83156338938584
H	-10.85067439915448	0.83859174906206	2.64453337712815
H	-11.55927296573321	3.43401664860620	0.24121567313418
H	-12.37986817999613	2.15487489797293	1.12027107147992
H	-11.43408880626819	2.01786550201929	-1.77943338417678
H	-13.08156613851025	2.27290631854628	-1.25111154187622
H	-13.07424804757908	0.03601175107163	-0.14992543198715
H	-11.42690299801048	-0.21986039254413	-0.67423826171517
H	-12.15122719591726	-0.04786784221052	-3.05204391557057
H	-13.80288967526506	0.19455789032731	-2.53213690675146
H	-13.78165705711434	-2.03961841992459	-1.42913382556491
H	-12.13129367164916	-2.28046819760607	-1.94925434585388
H	-14.52287595456491	-1.90746086341948	-3.82073572093374
H	-13.71004830274464	-3.43532479036823	-3.49304486375469
H	-12.86065335703203	-2.14870102040283	-4.34475723989417
H	7.11126216667751	-2.12498656220886	-1.98972683887378
H	7.22565898304816	-3.05567347462711	-0.50155218987882
H	7.55345651141401	-0.95398228537560	0.78974950747063
H	7.42242820777707	-0.01957947826224	-0.68992037696462
H	9.43150807568472	-1.18156306996445	-1.60189483307506
H	9.56465268069169	-2.11571324139258	-0.12712702981154

H	9.87704761131679	-0.01751862900536	1.17651428179322
H	9.73319725532163	0.92023382181184	-0.29483807082942
H	11.72893633335709	-0.22271508480337	-1.22699645392776
H	11.87736132010979	-1.16896720996673	0.24200376765174
H	12.15112412016300	0.90200642201354	1.58261621000848
H	11.94997241397972	1.88326022283498	0.14299609073923
H	13.89214411886293	2.99711865054977	0.60497973978143
H	15.41616797587989	2.12142949556152	0.86319674702018
H	14.11448403616957	2.00609036011392	2.07029035210327
H	14.09694545059608	-0.31799147632687	1.97831914825093
H	15.46341246623202	-0.22178829314395	0.84204894258992
H	14.02880274430490	-1.17547233585305	0.41989362434671
H	13.94540518076247	-0.05921016122157	-1.52797180597460
H	15.26495124930614	1.03619166591358	-1.14501951982265
H	12.44804008531486	1.89380043648708	-1.97274810924380
H	13.82102905915079	1.69639076692167	-3.03851827095857
H	14.79505521180295	3.42498850636317	-1.89614442352405

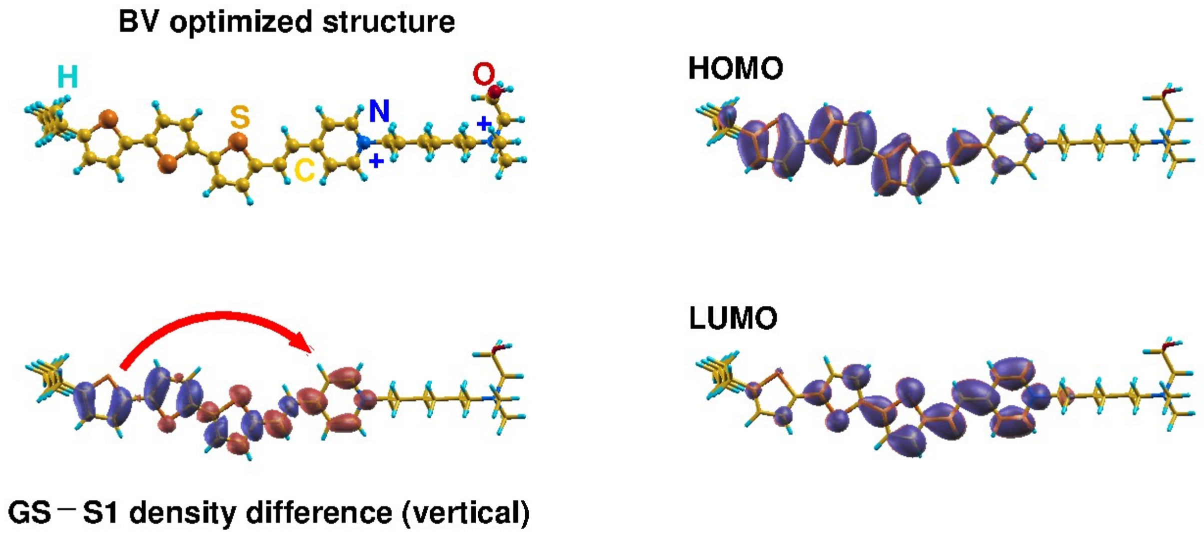

Figure S8

HOMO and LUMO orbitals of BV–1 calculated at the revPBE0@def2-TZVPP level of theory and density difference between the ground and S1 excited states in the geometry of the ground state (vertical). The red arrow indicates the displacement of charge density from blue to red regions upon GS→S1 excitation (charge transfer excited state).

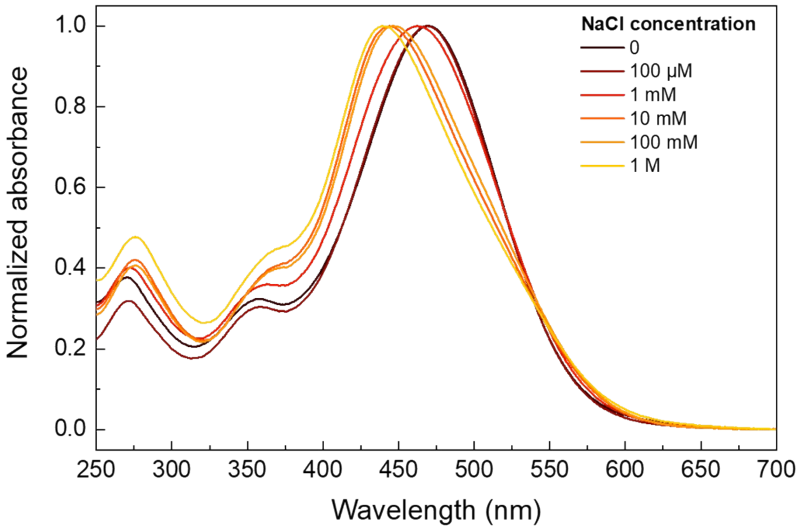

Figure S9

Normalized absorption spectra of BV–1 (25 μM) in NaCl aqueous solutions.

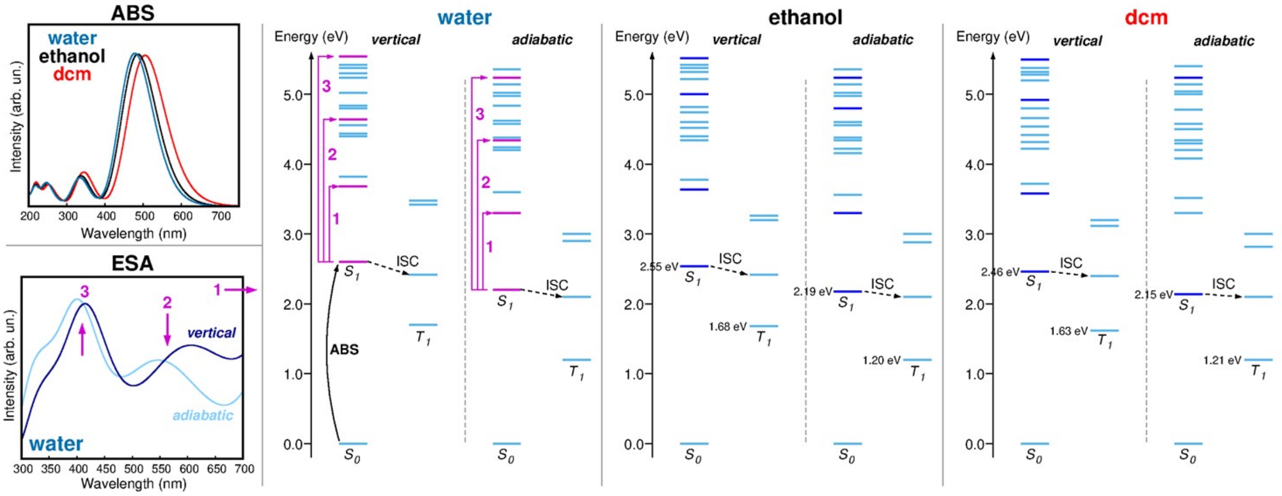

Figure S10

Jablonski diagrams of singlet and triplet excited states of BV–1 in vacuum and in solution (dichloromethane, ethanol and water). Vertical (calculated in the ground-state structure) and adiabatic (in the S1 excited-state structure) distributions are shown. Possible intersystem conversion (ISC) pathways are also indicated by dashed lines.

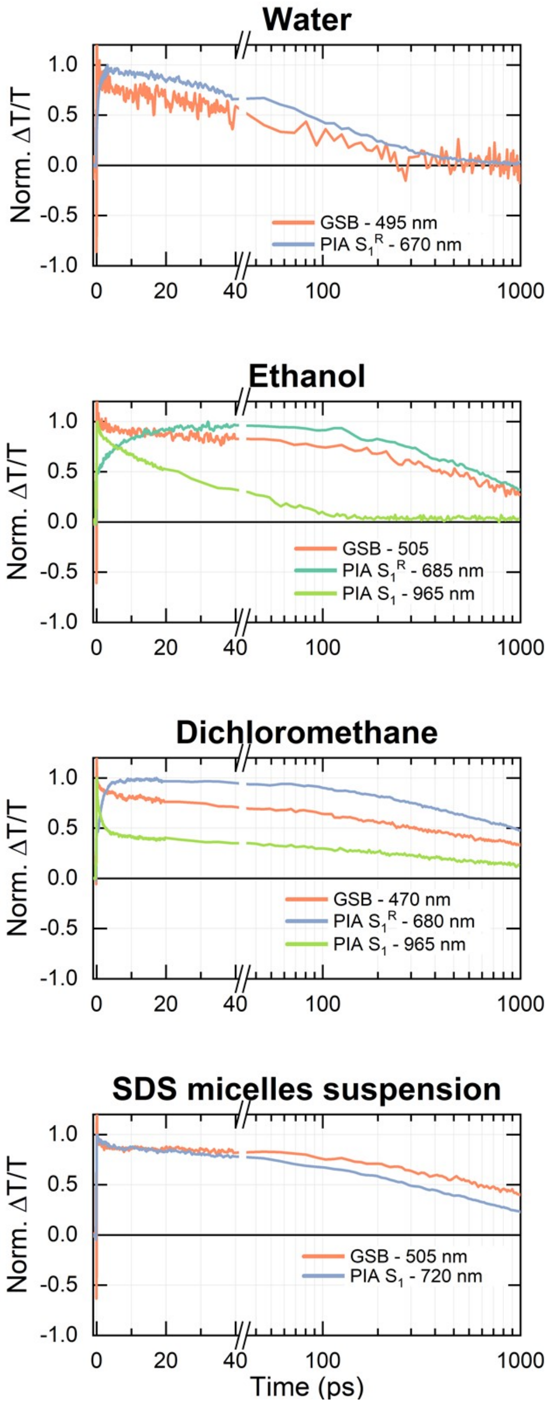

Figure S11

Transient absorption dynamics of BV–1 at different probe wavelengths.

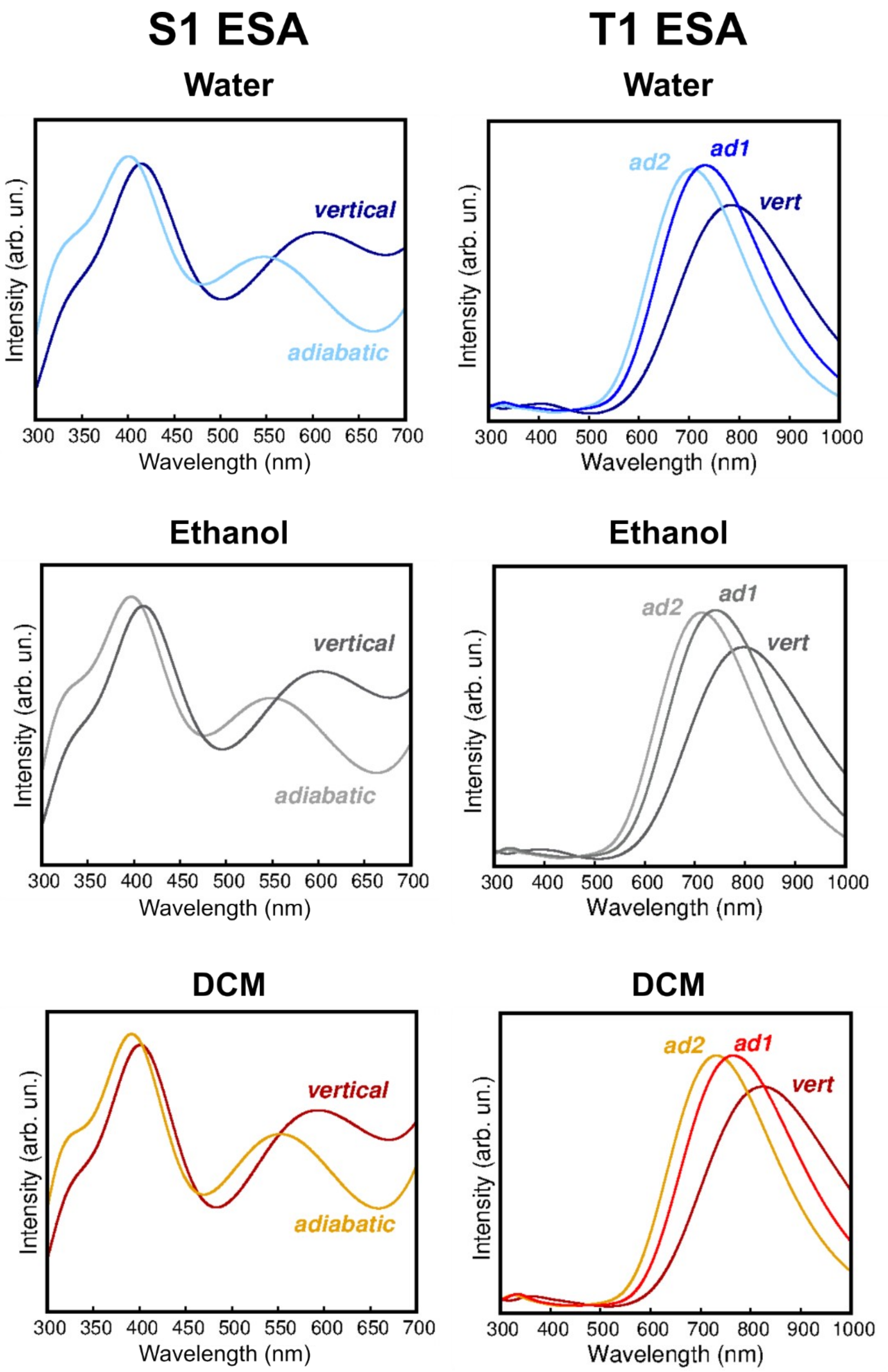

Figure S12

Excited-state absorption (ESA) from the S1 (left panels) and T1 (right panels) excited states in three solvents (dichloromethane, ethanol and water). ESA have been calculated both in the vertical (in the ground-state structure) and in the adiabatic (in the S1 excited-state structure for S1 ESA and in S1 and T1 structures for T1 ESA, labelled as ad1 and ad2, respectively) transition geometries. The vertical and adiabatic transitions can be interpreted as the limits of t=0 and long t transient absorption signals, respectively.

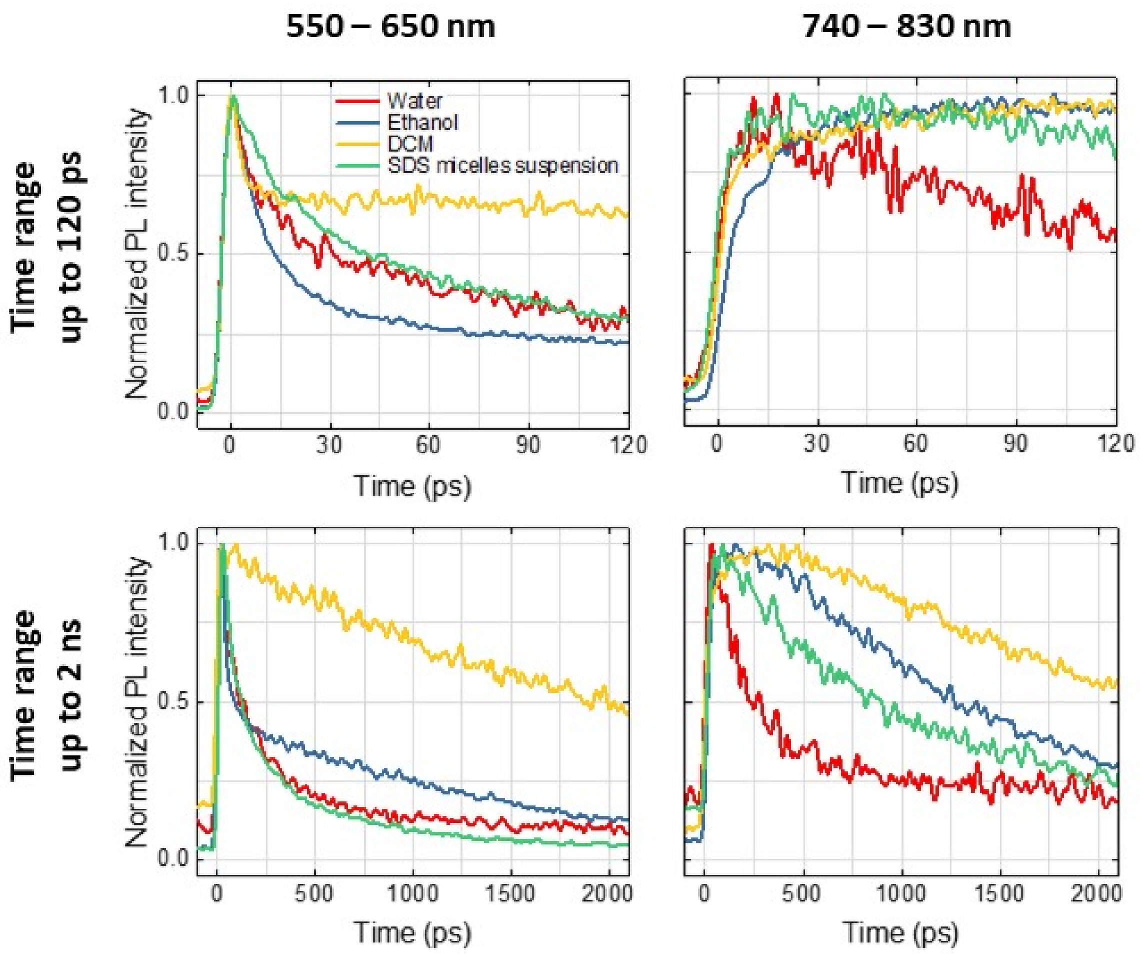

Figure S13

Fluorescence dynamics of BV–1 in different solvents (water, ethanol, dichloromethane, and SDS micelles suspension). The PL decays are plotted in two different spectral regions: 550-650 nm (left panels) and 740-830 nm (right panels). The lower the polarity of the solvent (DCM), the slower the PL decay. Moreover, at the longer wavelength, in ethanol and DCM we observe a slow rising of the PL signal, which is a hint of the building up of a population on a state different from the one reached by the molecule immediately after the excitation.

TRPL measurements were carried out using a femtosecond laser source coupled to a streak camera detection system (Hamamatsu C5680). A Ti:sapphire laser (Coherent Chameleon Ultra II, pulse bandwidths of 140 fs, repetition rate of 80 MHz, and maximum pulse energy of 50 nJ) was used to pump a second-harmonic crystal (β-barium borate) to tune the pump wavelength to 470 nm. The measurements here shown were performed recording the first 120 ps of decays, with an IRF of 4.1 ps (top panel) and recording the first 2 ns of decay with an IRF of ~30 ps.

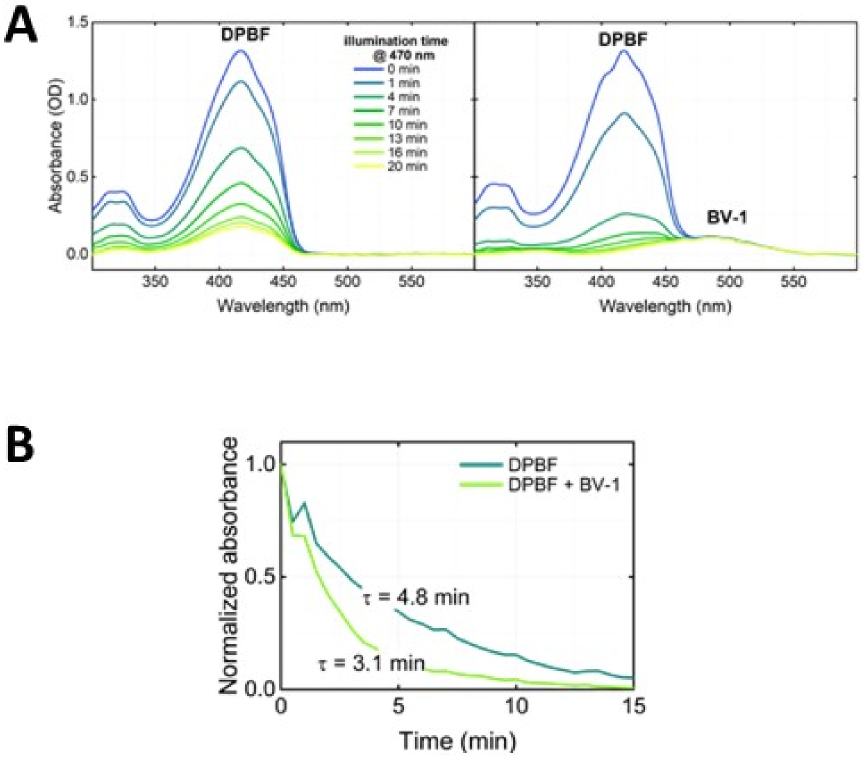

Figure S14

A. Time-evolution of the UV-VIS spectrum of a DPBF (50 µM) and of DPBF (50 µM) and BV–1 (5 µM) in DMSO solution. The samples were placed under a 470 nm LED light.

B. Normalized dynamics of the DPBF absorption peak (415 nm).

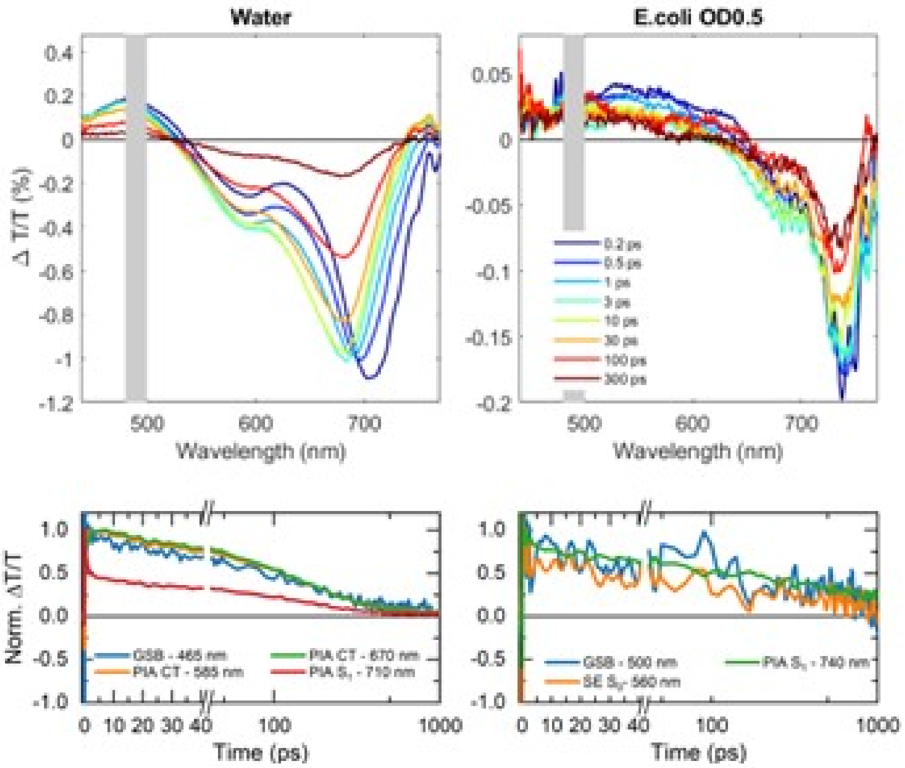

Figure S15

Transient absorption spectra and dynamics of BV–1 in water and in *E. coli* cells suspension. The system was excited at 490 nm and probed with two different white light super-continuums to probe both the visible and the NIR range. The solutions were flowed by means of a peristaltic pump, to avoid photobleaching of the samples.

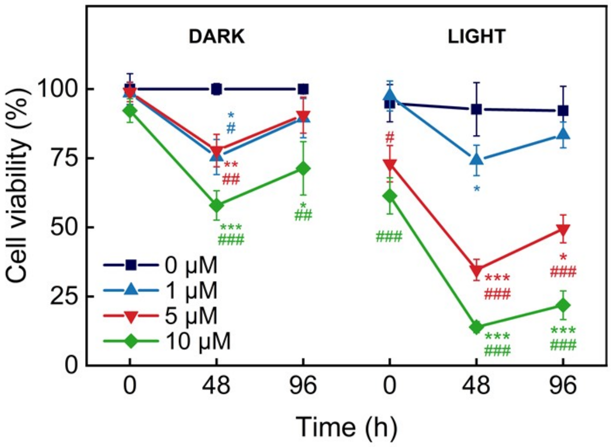

Figure S16

AlamarBlue viability assay. The points represent the average fluorescence intensity of AlamarBlue exposed to HEK-293T cells loaded with BV-1 at different concentrations (0, 1, 5 and 10 μM). The error bars represent the standard error of the mean (n=9 for all conditions). Each column of the graph reports the average over 3 distinct cell preparations. Statistical significance between two conditions was evaluated using two-way ANOVA-test. In all the reported data, $*p < 0.05$, $**p < 0.01$, and $***p < 0.001$. Stars (*) are used to show significance with respect to the values at time 0 h (same concentration), while hashes (#) are used to show significance with respect to the values at concentration 0 μM (at the same time point).

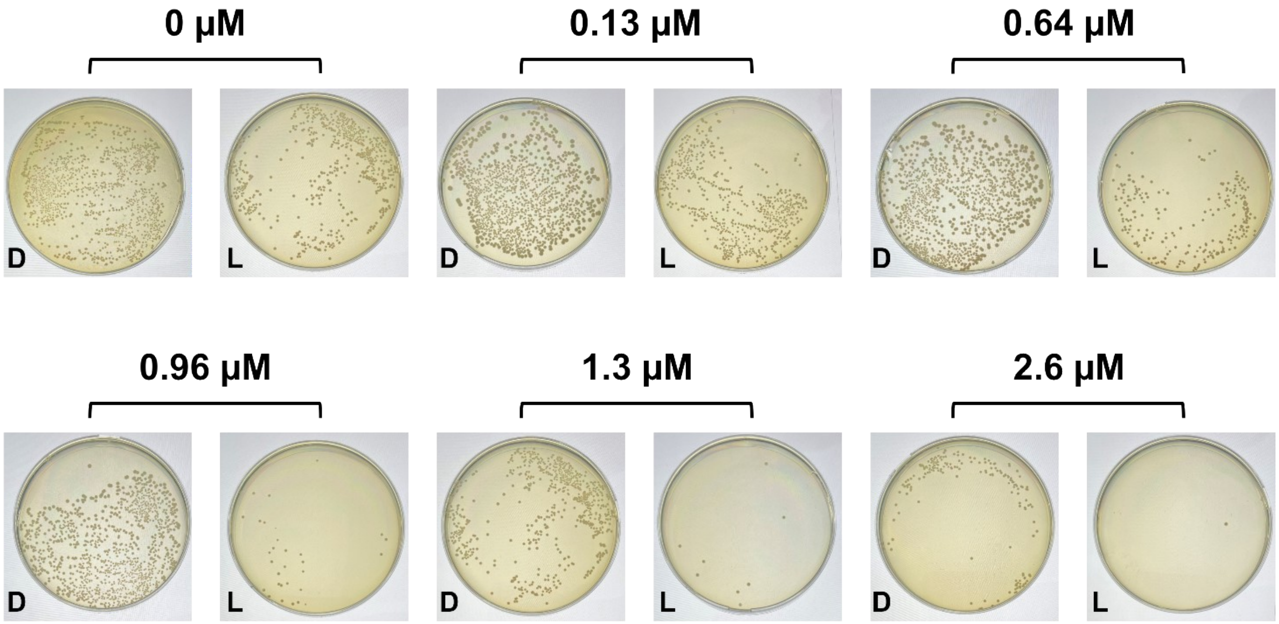

Figure S17

Representative pictures of *E. coli* colonies grown on LB agar plates after the exposure to BV-1 and grown under dark (D) or light (L) conditions as done in the MIC experiment.

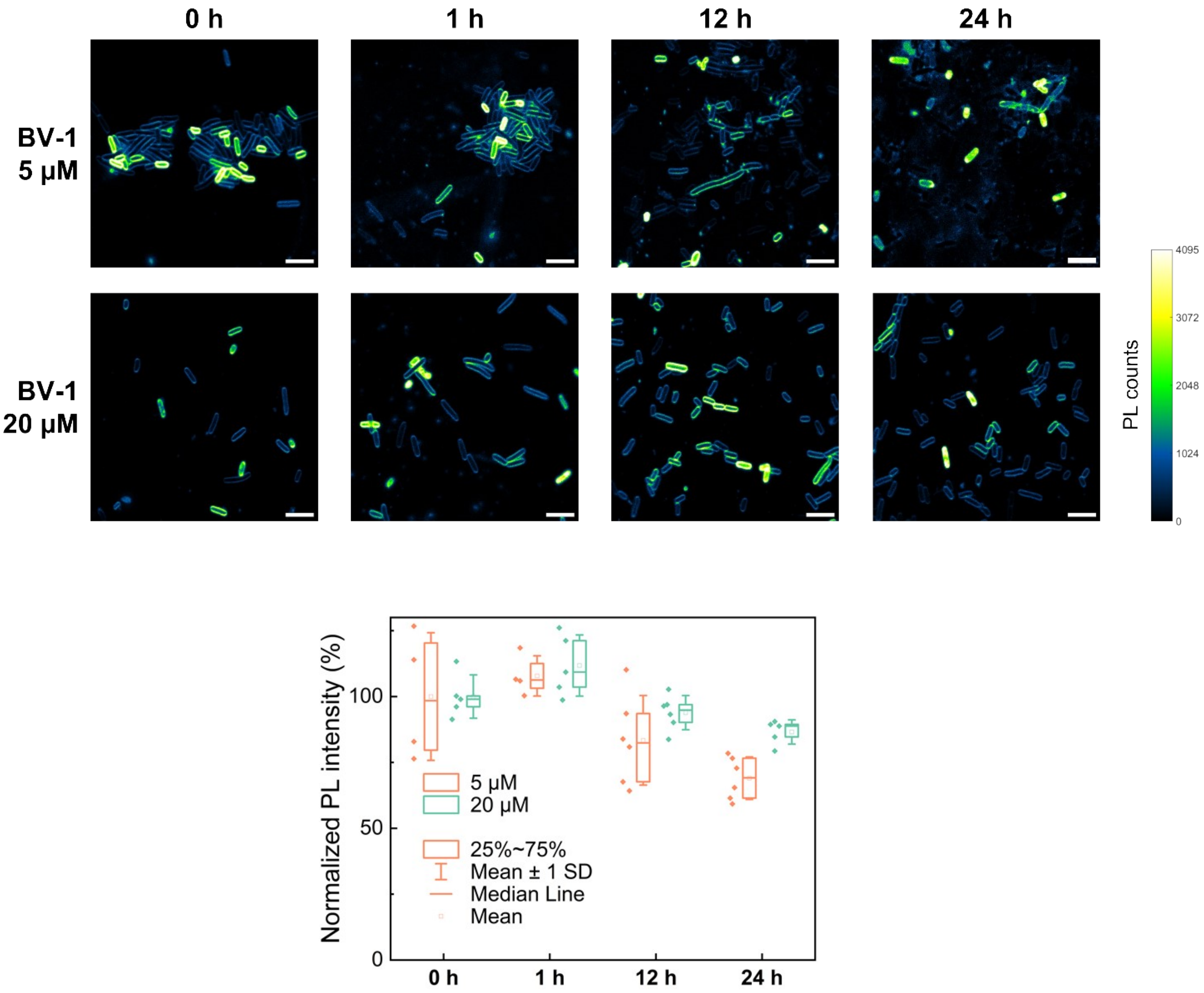

Figure S18

The top panel includes representative images of E. coli loaded with different concentrations of BV-1 (5 and 20 μM), taken at different time points after the treatment (scale bar 5 μm). The images show the fluorescence intensity. The bottom plot shows the PL intensity, normalized at time 0 in the two different conditions.

Rep

References

1. Neese, F. The ORCA program system. *WIREs Comput. Mol. Sci.* **2**, 73–78 (2012).

2. Neese, F. Software update: the ORCA program system, version 4.0. *WIREs Comput. Mol. Sci.* **8**, (2018).

3. Bannwarth, C., Ehlert, S. & Grimme, S. GFN2-xTB—An Accurate and Broadly Parametrized Self-Consistent Tight-Binding Quantum Chemical Method with Multipole Electrostatics and Density-Dependent Dispersion Contributions. *J. Chem. Theory Comput.* **15**, 1652–1671 (2019).

4. Grimme, S. Exploration of Chemical Compound, Conformer, and Reaction Space with Meta-Dynamics Simulations Based on Tight-Binding Quantum Chemical Calculations. *J. Chem. Theory Comput.* **15**, 2847–2862 (2019).

5. Pracht, P., Bohle, F. & Grimme, S. Automated exploration of the low-energy chemical space with fast quantum chemical methods. *Phys. Chem. Chem. Phys.* **22**, 7169–7192 (2020).

6. Zhang, Y. & Yang, W. Comment on “Generalized Gradient Approximation Made Simple”. *Phys. Rev. Lett.* **80**, 890–890 (1998).

7. Grimme, S., Antony, J., Ehrlich, S. & Krieg, H. A consistent and accurate ab initio parametrization of density functional dispersion correction (DFT-D) for the 94 elements H-Pu. *J. Chem. Phys.* **132**, 154104 (2010).

8. Schäfer, A., Horn, H. & Ahlrichs, R. Fully optimized contracted Gaussian basis sets for atoms Li to Kr. *J. Chem. Phys.* **97**, 2571–2577 (1992).

9. Cammi, R., Mennucci, B. & Tomasi, J. Fast Evaluation of Geometries and Properties of Excited Molecules in Solution: A Tamm-Dancoff Model with Application to 4-Dimethylaminobenzonitrile. *J. Phys. Chem. A* **104**, 5631–5637 (2000).

10. de Souza, B., Neese, F. & Izsák, R. On the theoretical prediction of fluorescence rates from first principles using the path integral approach. *J. Chem. Phys.* **148**, 034104 (2018).
